# Going With the Flow? Relative Importance of Riverine Hydrologic Connectivity Versus Tidal Influence for Spatial Structure of Genetic Diversity and Relatedness in a Foundational Submersed Aquatic Plant

**DOI:** 10.1002/ece3.71264

**Published:** 2025-05-07

**Authors:** Maile C. Neel, Brittany W. Marsden, Katharina A. M. Engelhardt

**Affiliations:** ^1^ Department of Plant Science and Landscape Architecture University of Maryland College Park Maryland USA; ^2^ Department of Entomology University of Maryland College Park Maryland USA; ^3^ Marine, Estuarine, Environmental Sciences University of Maryland College Park Maryland USA; ^4^ University of Maryland Center for Environmental Science, Appalachian Laboratory Frostburg Maryland USA

**Keywords:** clonality, resilience, restoration ecology, spatial genetic structure, submersed aquatic vegetation

## Abstract

Genetic connectivity in rivers is generally high, and levels of genotypic and genetic diversity of riverine species are expected to accumulate in downstream locations. Genetic structure of marine and estuarine species is less predictable, even though hydrologic connectivity is also expected to be relatively high in those ecosystems. These observations have been generated across different species and locations such that our understanding of the effects of hydrologic connectivity in the same river, spanning tidal and nontidal habitats, remains incomplete. To control for species and location, we quantified diversity in 941 samples of *Vallisneria americana* Michx. (Hydrocharitaceae) collected from 36 sites along the species' entire distribution in the tidal and nontidal Potomac River of Maryland, Virginia, and the District of Columbia, USA. Using 10 microsatellite loci, we found 507 unique multilocus genotypes (MLGs) that were collapsed to 482 multilocus lineages (MLLs). Fifty‐three MLLs were found multiple times across the riverscape, accounting for over 54% of the genotyped shoots. We found some evidence supporting connectivity throughout the river and stronger evidence that tidal regime drives genotypic and genetic structure within *V. americana* . Extensive clonality, including two MLLs spanning 230 and 152 km, limits diversity in the nontidal reaches and contrasts with very little evidence of clonal reproduction in tidal reaches. Genetic differentiation, structure, and pairwise relatedness of sampled shoots and MLLs also differed by tidal reach, with the nontidal Potomac having higher levels of relatedness, lower allelic diversity, and higher heterozygosity. The differences in spatial distribution of genetic diversity suggest very different outlooks for *V. americana* adaptation and acclimation to perturbations in tidal and nontidal regions of the Potomac, which lead to different recommendations for restoration of the same species in the same river.

## Graphical abstract

We assessed genotypic and genetic diversity in the submersed aquatic plant species *Vallisneria americana* in the Potomac River, USA, to understand how riverine hydrologic connectivity and tidal regime affected spatial genetic structure. We found striking differences in the structure of diversity, clonal growth, and relatedness in different tidal environments that override continuous hydrologic connectivity. These structural differences yield different outlooks for adaptation and acclimation and different management recommendations for the same species in different parts of the same river.

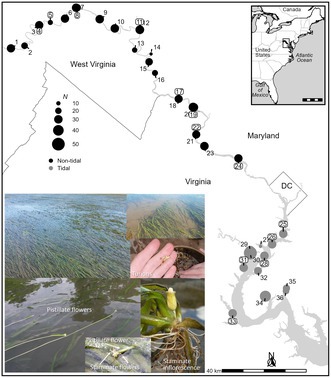

## 1 Introduction

Genetic diversity contributes to population resilience through increased fitness (Amos et al. 2001; Crnokrak and Roff 1999; Ellstrand and Elam 1993; Leimu et al. 2006; Williams 2001), enhanced growth and productivity (Reynolds, McGlathery, and Waycott 2012; Williams 2001), rapid response to disturbances (Hughes and Stachowicz 2004; Massa et al. 2013; Reusch et al. 2005), and greater persistence through time (Salo and Gustafsson 2016). The spatial structure of genetic diversity gives insight into the scales over which ecological processes of mating and dispersal drive the evolutionary processes that maintain, increase, or decrease diversity (Heywood 1991; Ouborg et al. 1999; Slatkin 1985). These scales differ among species as a function of how life‐history traits interact with the environment to enable persistence in and movement through landscapes (Manel et al. 2003). Understanding such interactions is critical for determining the distances over which diversity can be maintained through gene flow (Booth and Grime 2003; Lankau and Strauss 2007; Vellend 2006) and over which recolonization is likely to occur after disturbance. The differences in spatial distribution of genetic diversity ultimately provide insight into the overall health of populations and their ability to be resilient through adaptation or acclimation to current and future perturbations. Insights gained from quantifying spatial genetic structure can thus help natural resource managers anticipate the effects of environmental change and evaluate risks and benefits of alternative management and restoration strategies (Conceicao et al. 2024; Durka et al. 2017; Marsden et al. 2022; Mosner et al. 2012; Pometti et al. 2018; Shryock et al. 2017).

In aquatic plants, hydrologic connectivity facilitates gene flow and increases the scale of spatial genetic patterns relative to terrestrial systems (reviewed in Grosberg and Cunningham 2001). The dominant paradigm for rivers is weak overall genetic structure with a smooth continuum of declining similarity with distance (termed isolation by distance—IBD) along relatively continuous, linear habitat (Nilsson et al. 2010; Pollux et al. 2007). At the same time, the downstream flow of water is expected to favor unidirectional dispersal, resulting in limited genotypic and genetic diversity upstream and accumulated diversity downstream (Barrett et al. 1993; Geremew et al. 2018; Honnay et al. 2010; Liu et al. 2006; Love et al. 2013). The anticipated genetic patterns can be seen as an extension of Vannote et al.'s (1980) River Continuum Concept, which envisions rivers as longitudinally connected from headwaters to river mouth. However, downstream diversity accumulation is not ubiquitous (Gornall et al. 1998; Lundqvist and Andersson 2001; Pollux et al. 2007; Triest and Fenart 2014; Wubs et al. 2016). Deviations arise from discontinuous habitat (Smith et al. 2015); complexities such as bifurcating river confluences, waterfalls, or dams (Fausch et al. 2002; Ward et al. 2001); and upstream animal‐mediated dispersal (van Leeuwen et al. 2012). A second complicating factor is imposed when a portion of a river is tidally influenced. Hydrologic connections are less predictable in tidal waters where variable and bidirectional water flow and localized currents are expected to create complex and erratic spatial genetic structure among populations (Becheler et al. 2010; Jahnke et al. 2018; Johnson and Black 1984; Källström et al. 2008; Selkoe et al. 2010; Serra et al. 2010; Siegel et al. 2008; Sinclair, Krauss, et al. 2014; van Dijk et al. 2009). Mixed clonal and sexual reproduction, a life‐history trait common in aquatic plants (Eckert et al. 2016), is a third factor affecting the structure of genetic diversity (Arnaud‐Haond et al. 2020) and understanding how differing reproductive modes interact with tidal and nontidal environments to affect genotypic variation is of key interest.

**FIGURE 1:**
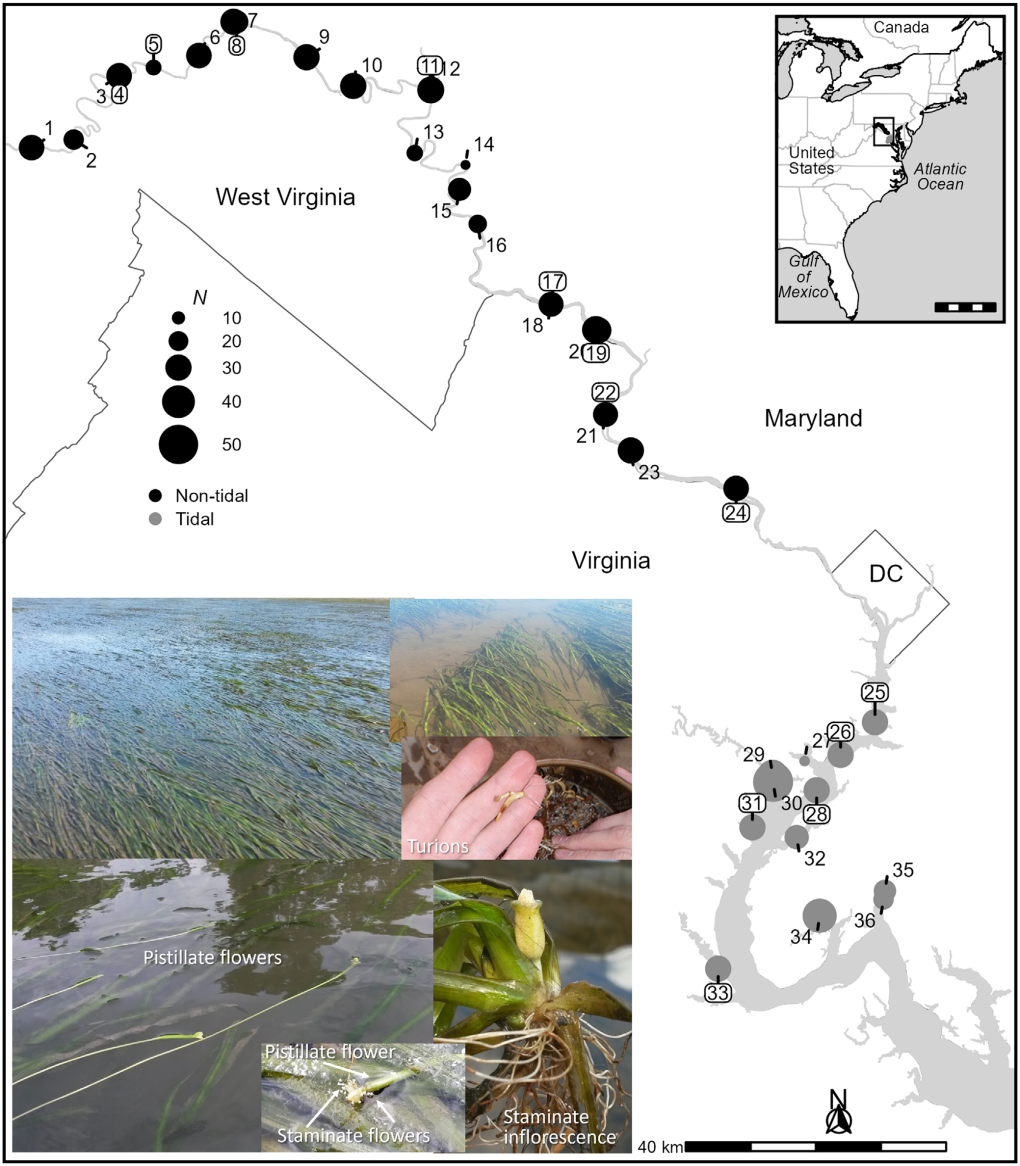
Thirty‐six *Vallisneria americana* sampling locations spanning the species distribution in the Potomac River, a major tributary of the Chesapeake Bay watershed. Site labels surrounded by a box are from Lloyd et al. (2011). The remaining sites are newly analyzed in this paper. Sample size (*N*) for each site is indicated by the size of the circle. Images show vegetative growth habit, pistillate (female) and staminate (male) inflorescences and flowers of *V. americana* . Photograph credits: Maile Neel. Political boundaries are from a dataset provided by the Commission for Environmental Cooperation (2022).

Although differences in genetic structure in riverine versus marine or estuarine environments are well documented, patterns have mostly emerged from studies of different species in disparate locations. As such, it is not possible to disentangle effects of life history and population history versus tidal regime. Here, we take advantage of a natural opportunity to begin to understand these relative influences by quantifying genetic structure of the submersed aquatic plant species *V. americana* Michx. (Hydrocharitaceae) collected from 36 sampling sites across its ~370 km distribution from nontidal fresh to tidal mesohaline waters in the Potomac River of Maryland, the District of Columbia, and Virginia, USA (Figure 1).

Previously, Lloyd et al. (2011) found that *V. americana* samples from eight nontidal Potomac River sites were somewhat distinguishable from five tidal sites based on Bayesian clustering of microsatellite allele frequencies; however, assignments of sampled shoots to theoretical population groups indicated extensive admixture in the vicinity of the transition from nontidal to tidal. They also saw little clonal growth in tidal sites in contrast with high clonality in nontidal sites, including four MLGs that spanned multiple nontidal sampling locations. Two of these MLGs each extended more than 100 km. No MLGs were shared between nontidal and tidal reaches or among tidal sites. However, the small number of tidal sites were farther apart than the nontidal sites, and it was not possible to be confident that the observed differences were not sampling artifacts. We expand on the original dataset by sampling 23 additional sites that span the full extent of *V. americana* in the Potomac River (Figure 1) using the same field and laboratory protocols. With these more extensive and finer grained samples, we ask if the levels and spatial structure of within‐site genotypic and genetic diversity and scale of gene flow among *V. americana* sites meet expectations of hydrologic connectivity (i.e., low differentiation, isolation by distance, and downstream accumulation of diversity) along the length of the river or if hydrologic connectivity is disrupted by tidal regime.

Answering these questions will allow us to understand if eco‐evolutionary dynamics in the two environments differ in ways that yield different outlooks for persistence and recommendations for management. Persistence of this species is a key conservation priority because submersed aquatic plants, such as *V. americana* , are foundation species that provide critical ecosystem services (Costanza et al. 1997), including improving water quality through sediment and nutrient sequestration (Gu 2008; Moore 2004; Takamura et al. 2003), physical sediment stabilization (Madsen et al. 2001; Sand‐Jensen 1998), and erosion reduction (Fonseca and Cahalan 1992). In addition, they promote diverse benthic communities (Orth, Harwell, and Inglis 2006), provide shelter and oxygenated habitat to nearshore fish communities (Findlay et al. 2006; Kemp et al. 2005; Strayer and Malcom 2007), and are a primary food source for waterfowl, fish, and invertebrates (Killgore et al. 1989; Orth, Carruthers, et al. 2006; Perry and Deller 1996). These far‐reaching ecological contributions make global declines of submersed aquatic plants a great concern and their conservation and restoration a priority (Orth, Carruthers, et al. 2006; Richardson et al. 2018; Waycott et al. 2009). The insights we gain from the spatial genetic structure of *V. americana* across two different but contiguous riverine environments will contribute to understanding its long‐term resilience as well as how well patterns and processes can be generalized across other locations and species in similar environments.

## 2 Methods

### 2.1 Study Species

*Vallisneria americana* is a perennial angiosperm that is native to habitats along the Atlantic coast of North America from southern Canada to Florida, along the Gulf coast to Texas, and north to the Great Lakes through major rivers (McFarland and Shafer 2008). Each spring, *V. americana* beds are formed by shoots that emerge from seeds or vegetative overwintering structures called turions (McFarland 2006). Each shoot has the capacity to produce more shoots through clonal expansion via stolons. Each individual, defined as a unique multilocus genotype (MLG) produces only male or female inflorescences (i.e., the species is dioecious; Titus and Hoover 1991; Figure 1). When female flowers are receptive to pollen, they float at the surface of the water but remain attached to the base of a maternal plant via a long peduncle. Minute male flowers break free from their inflorescence that grows at the base of a paternal plant, rise to the surface of the water, and float passively until they intercept a female flower (Figure 1). Due to the complexities involved, pollination typically occurs within 2–5 m of the paternal ramet (Lloyd et al. 2018). Even when males and females are equally abundant and in close proximity, opportunities for pollination can be limited by rapid currents that can hold female flowers underwater or carry male flowers out of beds and away from female flowers (Sullivan and Titus 1996). Near the end of a growing season, stolons shift their energy allocation to producing underground turions. After aboveground vegetation and stolons die back in the fall, seeds and physiologically independent clonal turions overwinter in the sediment. In the spring, the process of growth and reproduction repeats. There is as much variation in relative rates and spatial scales of sexual versus asexual reproduction across sites in this species (Lloyd et al. 2011; Marsden et al. 2022; Ngeve et al. 2023) as there is across species of angiosperms (Ellstrand and Roose 1987; Honnay and Bossuyt 2005; Triest and Fenart 2014; Widen et al. 1994).

### 2.2 Study Area

The Potomac River originates in West Virginia and flows ~652 km before discharging into the Chesapeake Bay at Point Lookout, Maryland. Draining a ~38,000 km^2^ watershed, it is the second largest tributary of the Bay, smaller than only the Susquehanna River (Mason and Flynn 1976). The first 464 km are nontidal, flowing through the Allegheny Plateau, Ridge and Valley, and Piedmont Provinces. The river then passes through a geologic feature called the fall line as its elevation drops quickly from the Piedmont to the Coastal Plain Province. The ~188 km between the fall line and the mouth of the river are tidal, with salinity ranging from freshwater (< 0.5 ppt), through oligohaline (0.5–5 ppt), to mesohaline (5–20 ppt) as one heads downstream (Carter and Rybicki 1986; Mason and Flynn 1976). Net flow is downstream (average annual flow = 323 m^3^ s^−1^; Carter and Rybicki 1986); however, flow in the estuary can be reversed during flood tides (Mason and Flynn 1976). Width of the nontidal portion of the river averages 200–400 m, narrowing to ~60 m at the fall line. Below that, the tidal Potomac rapidly broadens to ~1 km wide, reaching nearly 10 km across at its mouth (Mason and Flynn 1976). The Potomac River estuary is relatively shallow with a narrow 4–7 m deep channel flanked by extensive 1–2 m deep shoals on each shore (Carter and Rybicki 1986). *Vallisneria* is found along 246 km of the nontidal waters above the fall line and ~74 km below, extending into the mesohaline zone. High water flow rates and exposed bedrock substrate through the waterfalls of the fall line are not suitable for *V. americana* and contribute to a gap in samples along the river.

### 2.3 Collections

We collected 415 *V. americana* individuals from 16 nontidal sites in 2011, and 208 individuals from seven tidal sites in 2013 (Appendix A: Table A1, Figure 1). In the nontidal reaches, we surveyed at 8 km intervals in 2011 from Cumberland, downstream to Pennyfield Lock just above the fall line, collecting samples wherever they were present. We collected the 2013 tidal samples from all surveyed locations that supported plants. Sites supporting *V. americana* were in tributaries off the mainstem of the river. We analyzed these samples with 180 samples from eight nontidal sites and 150 samples from five tidal sites we had collected previously (Lloyd et al. 2011) for a total of 953 samples from 36 sites in the nontidal (*n* = 24) and tidal (*n* = 12) Potomac River. Combining all samples increased sample density and expanded to more closely encompass the upstream and downstream extent of the species in the river (Figure 1). The possibility of temporal bias is addressed in statistical analyses given below.

To be consistent with the sampling protocol of (Lloyd et al. 2011), we sought to collect ~30 shoots at each site at ~5–15 m intervals along transects parallel to river flow. Latitude and longitude were recorded for each sample using a handheld Garmin Etrex GPS unit. The actual number of shoots (range *n* = 5–51), and distances among them, reflected the distribution and density of plants encountered. Extremely small sample numbers from Site 1 (*n* = 5) and Site 27 (*n* = 6) included all shoots found despite extensive searches. At other sites, sparse distributions necessitated larger distances among samples. Tissue was placed on ice within 1 h of collection and kept cool during transport to the University of Maryland College Park.

### 2.4 DNA Extraction and Genotyping

Lloyd et al. (2011) used DNeasy Plant Mini Kits (Qiagen Inc. Germantown, Maryland, USA) to isolate DNA from samples. We isolated DNA from shoots collected in 2011 first using a Chelex Bead (Bio‐Rad Laboratories, Hercules, California, USA) extraction method, where a 1 cm^2^ fragment of leaf tissue was ground with a sterilized glass pestle in 200 μL of a 10% Chelex slurry. Samples were boiled at 100°C for 10 min on an MJ Research PTC‐200 Peltier Thermal Cycler. Supernatant containing DNA was removed and diluted 1:2 in sterilized deionized water. Due to excessive missing data from ~30% of the samples extracted with Chelex, we subsequently used sbeadx Plant Maxi DNA extraction kits (LGC Genomics, Beverly, Massachusetts USA) following the manufacturer's instructions to extract DNA from frozen tissue of the problematic samples. Samples from 2013 were extracted with Synergy 2.0 kits (OPS Diagnostics, Lebanon, New Jersey, USA) following the manufacturer instructions. All shoots extracted using both Chelex and LGC protocols were genotyped using both extractions, and the resulting genotype profiles were reconciled. Extracting samples from earlier collection periods with later extraction methods allowed us to ensure consistent allele calls across extraction protocols and observers. We found no differences in DNA quantity or quality or genotyping results among any of the kits, but found that the Chelex method did not provide reliable allele calls and recommended against its use.

From extracted DNA, we amplified 10 loci using primers and PCR protocols developed for the species (Burnett et al. 2009) and used in previous studies (Lloyd et al. 2011, 2018; Ngeve et al. 2023). PCR products were separated and measured on an Applied Biosystems 3730xl DNA Analyzer with a 500 LIZ Size Standard (Applied Biosystems/ThermoFisher Scientific, Waltham, Massachusetts USA). Peak data were analyzed using Applied Biosystems GeneMapper v3.7 software using the same bin definitions across all samples. Allele calls were visually inspected and made consistent with the previously analyzed data by following standards set by Lloyd et al. (2011) and Marsden et al. (2022). Samples with ambiguous allele calls were re‐genotyped, and calls that remained ambiguous after four attempts were coded as missing. Because we used the same protocols across time periods and we re‐genotyped a subset of earlier samples to calibrate genotype calls in later time periods, we are confident that the datasets can be combined.

### 2.5 Data Analysis

Except as noted, all analyses were conducted in R version 4.4.1 (R Core Team 2024).

### 2.6 Among‐Sample and Site Distances

Latitude and longitude of each sample were converted to Universal Transverse Mercator (UTM) coordinates in the NAD83 datum. Site centroids were calculated as the means of sample coordinates. Centroids for four sites fell on land and were moved to the closest location in water at the same position along the river gradient. We calculated the distance in kilometers from the mouth of the river (termed *river km*) for each site by projecting the population centroid to the midline of the river using ArcMap v10 (ESRI 2019). Sample locations ranged from river km 89 (Site 36) to km 462 (Site 1; Figure 1).

We calculated pairwise distances among all samples and site centroids using both Euclidean distance and cost distance restricted to water. Euclidean distance is more accurate at small spatial scales and when connections are direct paths because raster‐based distance calculations underestimate distance as a function of cell size. Cost distances are more accurate at larger spatial scales over which sites are separated by convoluted shorelines and circuitous river courses, and cell size is a negligible fraction of the total distance. Water extent was defined by digitizing the river shoreline following the satellite imagery in ArcMap (ESRI 2019). The resulting shapefile was converted to raster format with a 10 m cell size. The raster file was read into R using the function *rast* from the terra package (Hijmans 2024) and was corrected to ensure that all sample points and site centroids fell within cells coded as water. We converted the corrected raster to a transition matrix using the gdistance package version 1.6.4 (van Etten 2017) with the *transition* function defined as the minimum cost between the eight neighboring cells with correction for differences in edge‐to‐edge versus corner‐to‐corner connections. We assigned water a conductance of one and land a conductance of zero (i.e., it was impassable). Shortest path cost distances among all pairs of samples and site centroids were calculated using the *costdistance* function by total cost of cells traversed in the transition surface by the cell size. To get the most accurate distance, we used the larger of Euclidean and cost distances for each pairwise comparison of samples and sites (as in Ngeve et al. 2023).

### 2.7 Identifying Multilocus Genotypes

Shoots were first assigned to MLGs based on complete multilocus matches. Shoots collected in or before 2011 were assigned with Genodive v2.0b17 (Meirmans and Van Tienderen 2004). The 2013 samples were assigned using the *mlg* function in the R package poppr Version 2.9.6 (Kamvar et al. 2015, 2014) and integrated into the Lloyd et al. (2011) MLG assignment framework. To determine if new collections were the same as existing MLGs, we assigned samples from all years to MLGs and filtered for MLGs with samples from multiple years. In such cases, the new samples were assigned the original MLG code. Each new MLG was assigned a unique code.

After the initial assignment of MLGs, we investigated any potential errors in estimating genotypic diversity with additional analyses using functions in the aforementioned poppr package. Requiring complete matches prevented underestimating genotypic diversity but assigned 58 shoots with missing allele data to distinct MLGs. To prevent overestimating the number of MLGs, we manually checked all shoots that had missing data and assigned them to new MLGs only if they were unique based on resolved loci. Next, we plotted the distribution of genetic distance among MLGs using the function *filter_stats* to identify similar MLGs that could represent potential scoring errors or somatic mutations (following Arnaud‐Haond et al. 2020). We used that distribution to identify the distance threshold below which MLGs were further evaluated for collapsing into multilocus lineages (MLLs). We did this collapsing using the function *mlg_filter*, when the differences included only one allele and locus, and if the less common MLG did not occur more than once. We assessed the power of the microsatellite data to distinguish among the original MLGs and the MLLs using the *genotype_curve* function. Finally, for MLLs occurring more than once, we calculated *psex* (Arnaud‐Haond et al. 2007; Parks and Werth 1993) using the multiple method to assess the probability of seeing the MLL the number of times it was observed, if those instances arose from separate zygotes. We considered additional occurrences of an MLL beyond the first to be a member of the same clone if *psex* was < 0.001, an arbitrary but reasonably low probability of seeing multiple instances of a genotype by chance if it was not due to the same sexual reproductive event.

### 2.8 Quantifying Genotypic Diversity

To understand the magnitude of asexual reproduction, we counted the number of single‐stemmed versus multistemmed MLLs across the entire sampling extent, in each tidal regime, and in each of the 36 sample sites. We calculated the extent of each multistemmed MLL as the number of times it occurred and as the maximum distance among samples through water, paying particular attention to MLLs that occurred across multiple sites. We also noted MLLs that were detected in different years as an indication that we were calling alleles consistently through time.

We used the poppr *mll* function to calculate the number of MLLs, as well as the Shannon and Gini‐Simpson diversity indices for the entire river, each tidal regime, and each site. For each sampling scale, we standardized the index values as the exponent of Shannon's index (Eff_Shannon_) and the reciprocal of Simpson's index (Eff_Simpson_). These standardizations give the effective number of MLLs, defined as the number of equally abundant MLLs that would yield the observed index value (Jost 2007). Effective numbers overcome the nonlinear behavior of the raw indices (Jost 2007), and because they are expressed as numbers of MLLs, they are intuitive to interpret and compare. The relative decline from the number of MLLs to Eff_Shannon_ to Eff_Simpson_ highlights evenness versus dominance. If all samples are different MLLs, they are equally frequent and all effective numbers will be the same maximum value. As MLL abundances become increasingly uneven, the three effective numbers decline in a predictable order, and differences among them increase due to differential sensitivity to influence rare versus common genotypes. We used Pareto *β* as an alternative approach to ranking populations in terms of unevenness among MLLs (Arnaud‐Haond et al. 2020; Stoeckel et al. 2021).

We calculated genotypic richness (*R*) as (*G −* 1)/(*N −* 1), where *G* is the number (or effective number) of MLLs in *N* genotyped shoots (Arnaud‐Haond et al. 2007) at the three sample scales (river, tidal regime, sites). *R*, *R*_Shannon_, and *R*_Simpson_ will be one if all samples are unique genotypes and zero if all samples are the same genotype and will decline monotonically according to the sensitivity of the respective effective numbers to rare versus common MLLs. We also calculated all genotypic diversity measures with samples rarefied to our smallest sample size (*n* = 5) to control for sample size differences.

We used the *coancestry* function in the R package‐related version 1.0 (Pew et al. 2015) to estimate pairwise relatedness among all samples and among distinct MLLs using Wang's *r* (Wang 2002). Relatedness among all samples gives insight into the combined influence of clonal and sexual reproduction on relationships among shoots. High values result if many shoots are the same MLL or if different MLLs are closely related to one another. Wang's *r* among only distinct MLLs tells us the unique component of relatedness due to sexual reproduction among relatives. We chose Wang's *r* because the *compareestimators* function indicated low variance and bias across relationship categories when analyzed using procedures of Taylor (2015). To determine if relatedness differed from expectation across the entire Potomac River or within tidal regimes, we used the *grouprel* function to compare observed values with distributions of values generated by randomly shuffling samples between the groups 1000 times while keeping each group size constant. Additionally, we calculated the mean pairwise Wang's *r* of each sample to all other samples within the same site (*r*_*W*_) and to samples from other sites within the same tidal regime (*r*_*A*_). We then summarized *r*_*W*_ and *r*_*A*_ to the site level as the average of all samples in each site. We followed the same process using one representative of each MLL per site.

### 2.9 Measures of Genetic Diversity

We calculated standard within‐site measures of genetic diversity at each of the three sampling scales. In each case, we used all samples to avoid bias introduced by removing clonal replicates (as suggested by Meirmans 2024). The number of polymorphic loci (P) and alleles per locus (*A*) were obtained from the summary of the genind object. The number of alleles per locus rarefied to the smallest number of alleles (*n* = 15; *A*_*r*_) was estimated using the *allelic.richness* function in the hierfstat R package version 0.5‐11 (Goudet 1995). Mean observed (*H*_*o*_), expected (*H*_*s*_) heterozygosity, and the deviation from the ratio of *H*_*o*_ *and H*_*s*_ expected under Hardy–Weinberg equilibrium (*F*_IS_) across loci were calculated using the hierfstat function *basic.stats*. We considered *F*_IS_ significant when the 95% confidence intervals calculated from using 1000 bootstrap replicates in *boot.ppfis* did not include zero. Variance across loci for F_IS_ was calculated as it is suggested to give insight into rates of clonality (Stoeckel et al. 2021). We also calculated site‐levels *H*_*o*_, *H*_*s*_, and *F*_IS_ using clone correction (i.e., with one instance of each MLL in each site) to compare with values from all samples and used the differences between the estimates to assess clonality (Meirmans 2024).

#### 2.9.1 Genetic Structure Among Sites and Tidal Regimes

We used all samples to summarize among‐site genetic diversity using Wright's *F*_ST_ (Weir and Cockerham 1984), $G_{\mathrm{ST}}^{\prime \prime }$ (Hedrick 2005), and *D*_est_ (Jost 2008). We report *F*_ST_ for comparative purposes because it is the most commonly used measure of among‐population genetic variance. However, it is downward biased with highly heterozygous microsatellite markers (Bossart and Pashley Prowell 1998; Hedrick 2005; Neigel 2002). We calculated $G_{\mathrm{ST}}^{\prime \prime }$, a version of *G*_ST_ scaled to the maximum possible for the observed amount of heterozygosity (Hedrick 2005). We calculated *D*_est_ because it describes genetic differentiation based on the effective numbers of alleles, which overcomes limitations of heterozygosity‐based measures (Jost 2008; Meirmans and Hedrick 2011). All three measures of among‐population genetic variance were calculated globally and pairwise for each site overall, each site within tidal regime, and for the two tidal regimes. For global measures, we used the hierfstat function *wc* for *F*_ST_ and the MMOD version 1.3.3 (Winter 2012) function *diffstats* for $G_{\mathrm{ST}}^{\prime \prime }$ and *D*_est_. The global measures were considered to be significant when 95% confidence intervals from 1000 bootstraps did not include 0. Bootstrap estimates for *F*_ST_ were done with hierfstat's *boot.vc* function. For the MMOD estimators, we generated bootstrap samples with *chao_bootstrap* and generated $G_{\mathrm{ST}}^{\prime \prime }$ and *D*_est_ for those samples using *summarize_bootstrap* in MMOD. Paired comparisons were calculated using *pairwise.WCfst* from hierfstat, and *pairwise_Hedrick* and *pairwise_D* from MMOD.

We used Mantel tests as implemented by the *mantel.randtest* function in the R package adegenet version 2.1.10 (Jombart and Ahmed 2011) to test for IBD among sites based on the magnitude and significance of the correlation between geographic distance and Edward's chord genetic distance. We calculated Edward's chord distance with the *dist.genpop* function in adegenet using all samples. To understand how tidal regime affected IBD, we conducted three tests: one using all sites and one for sites within each tidal regime. We assessed the probability of seeing the relationships in each comparison by random chance using 999 permutations.

Finally, we used correspondence analysis (CA) as implemented by the *CA* function in the R package FactoMineR version 2.1.1 (Lê et al. 2008) to understand relationships among sites based on a two‐way contingency table of allele frequencies (Kostov et al. 2015). In CA, contingency tables are reduced to fewer dimensions to capture the majority of allelic variance and identify associations between sites and alleles. When these dimensions are plotted, sites with similar allele frequencies are placed close to one another. If genetic variation is primarily due to geography, we expect sites in the ordination space to reflect their distribution along the river. If combining samples from different years over the 6‐year time period affected our results, we would expect samples from the year of sampling to override geography.

### 2.10 Testing for Differences Among Sites in Different Tidal Regimes

To quantify the probability of seeing the observed differences in site‐level measures of genotypic diversity, relatedness, and genetic diversity between tidal regimes by chance, we permuted values across sites 10,000 times using the *permutations* function in the modelr package version 0.1.11 (Wickham 2023). We calculated the absolute value of the difference between the mean in each tidal regime for each permutation and the observed data. The proportion of permuted differences that were larger than the observed was recorded as the probability of the observed value occurring by chance. Nonsignificant values are reported to the second decimal place. We tested for accumulation of diversity along the Potomac River using Spearman rank correlation analysis of each measure of genotypic and genetic diversity against river km as implemented in the function *rcorr* in the package Hmisc version 5.1–3 (Harrell Jr. 2017).

## 3 Results

### 3.1 Geographic Distances Among Samples and Sites

Nearest neighbor distance for each sample ranged from 0 m (below the resolution of the GPS unit) to 137 m with an overall median nearest neighbor distance of 10.1 m. The median of minimum distances calculated among samples within sites ranged from 4.2 to 27.9 m (Appendix B: Table A2). Site extents (maximum distances among samples within sites) ranged from 66.6 to 4652 m (Appendix B: Table A2). The large spatial extents at four tidal sites reflect the relatively sparse but widespread distribution of the species in some tidal tributaries. Even within these sites, some nearest neighbor distances among samples were similar to the more typical sites.

Nearest neighbor distances among sites based on the larger of Euclidean or cost distance within water ranged from 0.1 to 24.1 km with a median of 6.5 km. Among nontidal sites, nearest neighbors ranged from 0.1 to 19 km, with a median of 3.8 km; these same values for tidal sites were 1 to 24.1 km, with a median of 6.8 km. The largest distance through water between pairs of sites was 380.0 km and, on average, the median distance of one site to all other sites was 129.4 km. The most distant tidal sites were 78.7 km apart, and the median distance from a site to all others averaged 31.3 km. In the nontidal, these distances were 193 and 82.2 km, reflecting the greater extent of the nontidal region. The gap between tidal and nontidal samples includes the fall line where the river passes from the Piedmont Province to Coastal Plain Province as well as a tidal portion of the River. The rocky substrate and fast currents through the fall line make it unsuitable habitat. We know of historic *V. americana* locations in between the fall line and our most upstream tidal locations. Despite extensive field surveying including in and around historic locations, we have found no beds of *V. americana* below the fall line upstream of Site 25. Additional sampling is needed to confirm that the gap reflects the distribution of *V. americana* in the river.

### 3.2 Data Quality and Genotype Assignments

Our dataset contained 953 samples (595 nontidal and 358 tidal), including the 623 samples we collected in 2011/2013 and 330 samples collected in 2007/2008 by Lloyd et al. (2011). The nontidal samples collected in 2007/2008 versus 2011 showed similar levels of genotypic diversity, and the same dominant clones were detected in both time periods. Levels of diversity and differentiation were also similar among tidal sites, regardless of whether they were sampled in 2007/2008 or 2013.

**FIGURE 2:**
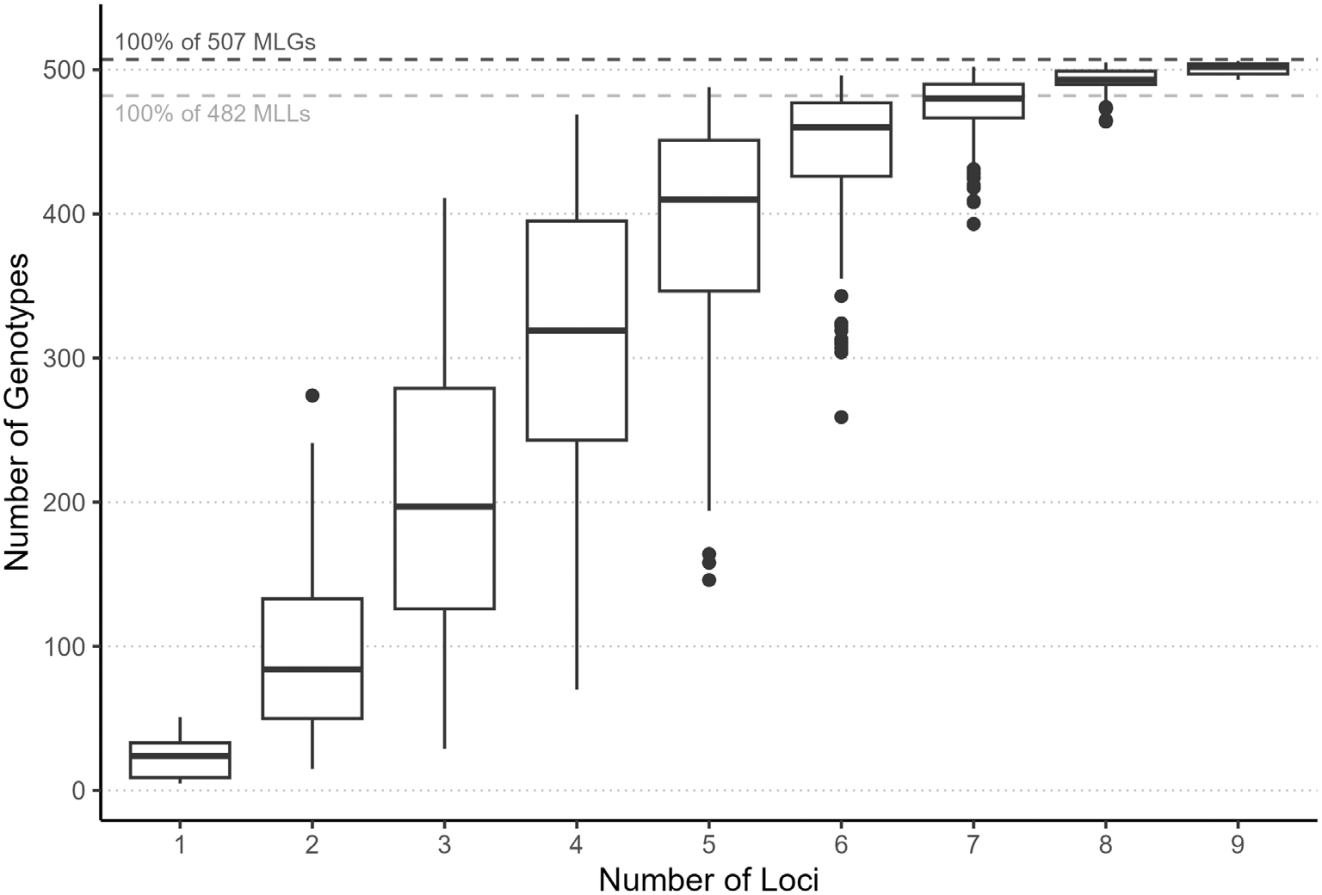
Accumulation curve showing power of different numbers of loci for detecting all 507 multilocus genotypes (MLGs) and 482 multilocus lineages (MLLs).

Genotyping resulted in 0.1% and 1.4% missing data per locus in nontidal and tidal samples respectively, with 59 samples having missing allele information at one or more loci. Missing data precluded unambiguous assignment of 13 nontidal samples to MLGs. The remaining 46 samples with missing data were unique based on the resolved loci and were assigned as MLGs, yielding 941 shoots assigned to 507 MLGs (187 nontidal, 320 tidal). Collapsing MLGs with small genetic distances reduced the MLGs to 482 MLLs (173 nontidal, 309 tidal). Although all loci were needed to distinguish all MLGs, the genotype accumulation curve indicates very small incremental increases in MLGs after seven loci (Figure 2), with only 1% of MLGs added at the final step. Values of *psex* for MLLs that occurred multiple times were always < 0.001. This low probability of observing replicate occurrences from different sexual reproductive events by chance gave us confidence that they are members of the same clonal lineage.

### 3.3 Genotypic Diversity

River‐wide, the 482 MLLs (*R* = 0.51) yielded Eff_Shannon_ of 99 (*R*_Shannon_ = 0.10), Eff_Simpson_ of 11.7 (*R*_Simpson_ = 0.01), and Pareto *β* was 0.15. The extremely small effective numbers of MLLs were driven by lower diversity and higher dominance in the nontidal regime where 582 samples yielded only 173 MLLs (*R* = 0.30). This unevenness was reflected in Eff_Shannon_ of 17.8 (*R*_Shannon_ = 0.03), Eff_Simpson_ of 4.5 (*R*_Simpson_ = 0.01), and Pareto *β* of 0.10. By contrast, 358 tidal samples yielded 309 MLLs (*R* = 0.86), Eff_Shannon_ of 234.8 (*R*_Shannon_ = 0.79), and Eff_Simpson_ of 234.7 (*R*_Simpson_ = 0.65), and Pareto *β* of 1.82. Overall, *R*_Shannon_ diversity was 18% of the river‐wide value of *R*, 10% of total *R* in the nontidal sites, and 92% of *R* in the tidal sites. *R*_Simpson_ was 2%, 3%, and 76% of the *R* in the full river, nontidal, and tidal reaches, respectively.

**FIGURE 3:**
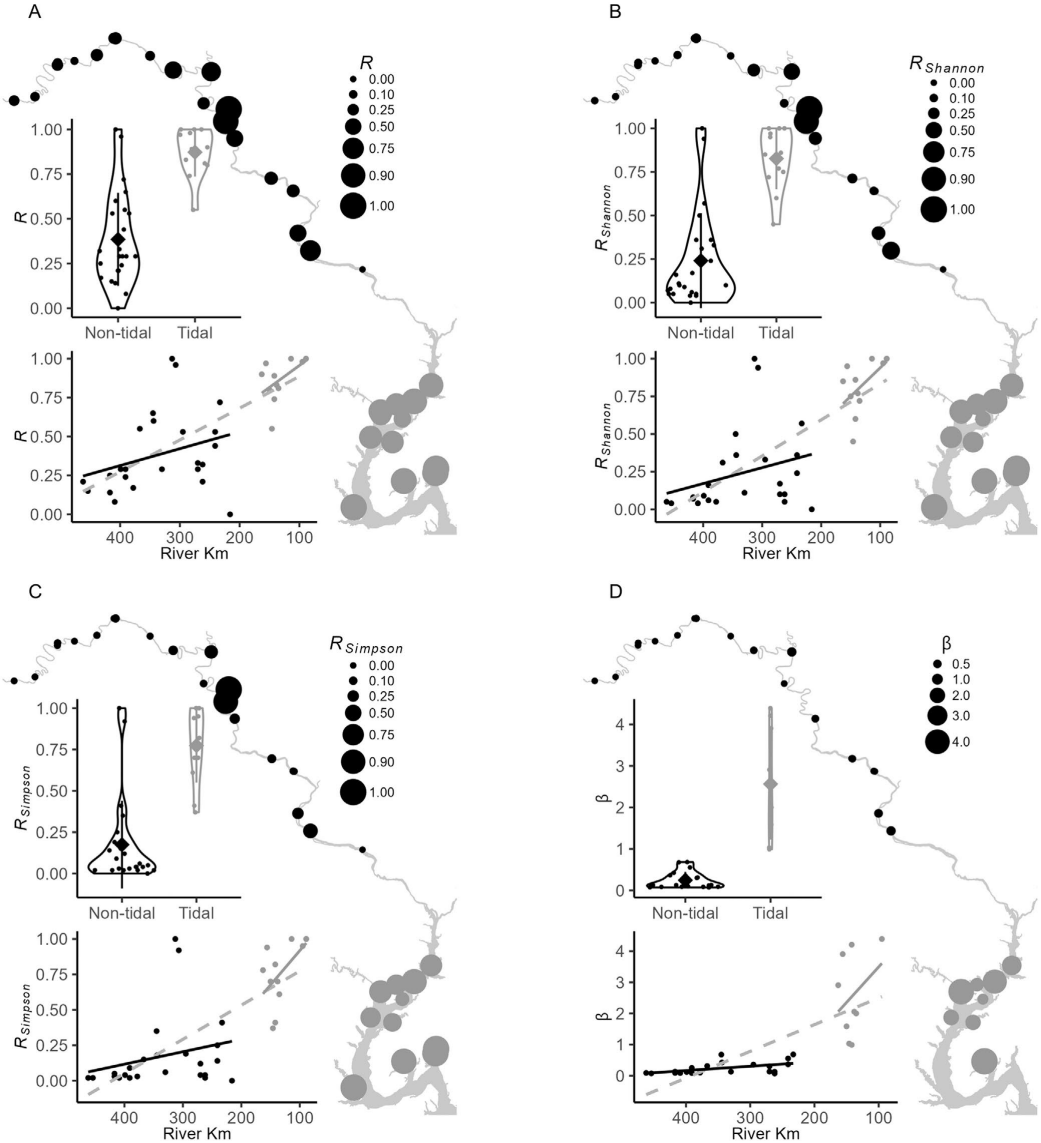
Site‐level genotypic richness (*R*) across the entire sample region of the Potomac River from nontidal (black circles) and tidal (gray circles) based on (A) the number of multilocus genotypes (MLLs), and the effective numbers of MLLs calculated from standardized (B) Shannon's index, (C) Simpson's index, and (D) Pareto *β*. Nontidal and tidal reaches are compared statistically using violin plots and correlations of *R* with river kilometer within each tidal reach (solid lines) and across the entire river (gray dashed line). Mean values among samples are indicated by the gray diamonds and error bars are one standard deviation.

Within the 36 sites, we genotyped on average 26.1 (SD = 8.9) shoots, yielding between 1 and 41 MLLs and *R* ranging from 0.00 to 1.00 ($\bar{x}$ = 0.55, SD = 0.32; Figure 3A). *R* in nontidal sites ranged from 0 to 1, but ranged only from 0.55 to 1 in tidal sites, resulting in lower mean *R* in nontidal sites ($\bar{x}$ = 0.38, SD = 0.26) than in tidal sites ($\bar{x}$ = 0.87, SD = 0.14; *p* < 0.0001; Figure 3A). Further, comparison of effective numbers of genotypes within sites revealed greater dominance by fewer genotypes in the nontidal than in the tidal sites, with the observed values being more extreme than all 10,000 permutations in all cases. *R*_Shannon_ diversity averaged 60% of total *R* in the nontidal sites and 94% of *R* in the tidal sites (Figure 3B,C). *R*_Simpson_ averaged 45% versus 88% of the *R* in the nontidal and tidal sites, respectively. Pareto *β* averaged 0.25 in nontidal sites and 2.6 in tidal sites (Figure 3D).

**FIGURE 4:**
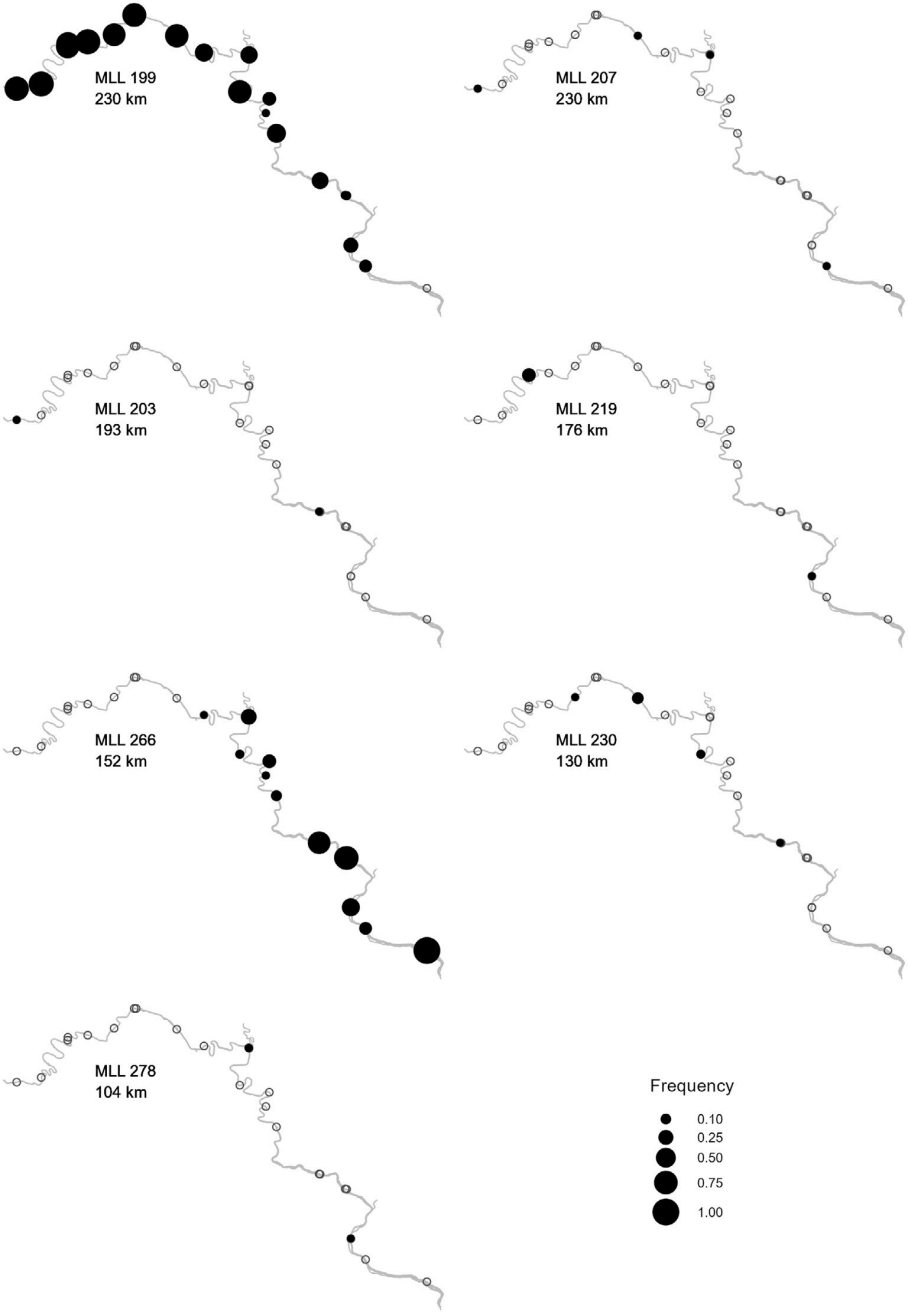
Distribution of seven *Vallisneria americana* multilocus lineages (MLLs) that occur at multiple nontidal collection sites in the Potomac River. Sites with no instance of a given MLL are indicated with open circles.

Unevenness of MLLs was driven by 53 MLLs that were found multiple times, accounting for 54.4% of all shoots. Twenty‐nine multisample MLLs were from the tidal, where they represented 9.4% of the MLLs and 21.8% of the tidal shoots. The 24 multisample MLLs from the nontidal represented 13.9% of the MLLs, but 74.4% of the nontidal sampled shoots. These nontidal MLLs extended from < 0.5 m to 233 km, with 17 occurring at multiple sites, and eight of those extending > 100 km (Figure 4). Two nontidal MLLs (MLL199 and MLL266) were particularly widespread and dominant (Figure 4). MLL199 was sampled 236 times from 23 sites spanning ~230 river km, representing 4%–86% of shoots genotyped where it was found. We found MLL266 137 times in 15 sites spanning 152 river km, representing 4%–100% of shoots genotyped in those sites (Figure 4). By contrast, only three tidal MLLs were sampled from more than one site. Two of these were also unique MLGs, both of which were shared by the two adjacent sites in Belmont Bay. Specifically, MLL502 was found eight times, once in Site 29 and seven times in Site 30, and MLL 3337 was found once in Site 29 and twice in Site 30. Neither of these MLLs extended more than 1.5 km. MLL 543 was found 20.3 km apart in Sites 26 and 31. This MLL represented two MLGs that differed by one locus. The 26 other tidal MLLs that occurred multiple times were restricted to single sites, where they comprised 3.9%–33.3% of the genotyped shoots, with spatial extents ranging from ~1 to 186 m.

Number of MLLs (*ρ* = −0.67, *p* = 6.8*e* ^−6^) and *R* (*ρ* = −0.73, *p* = 4.7*e* ^−7^) were negatively correlated with river km, indicating downstream accumulation of genotypic diversity. However, the correlation was driven primarily by the higher *R* in tidal than nontidal sites. When they were examined separately, correlations with *R* were much weaker and were marginally significant (tidal *ρ* = −0.58, *p* = 0.048; nontidal *ρ* = −0.40, *p* = 0. 051; Figure 3A). Correlations between the number of MLLs and river km were not significant in either (tidal: *ρ* = −0.07, *p* = 0.837; nontidal: *ρ* = −0.33, *p* = 0.115). Correlations of *R* and river km based on both effective numbers of genotypes mirrored standard *R* river‐wide but were not significant within either tidal regime (Figure 3B,C). Pareto *β* was lower upstream in the whole river (*ρ* = −0.83, *p* = 1.3*e* ^−8^) and in nontidal (*ρ* = −0.57, *p* = 0.007) but not in tidal sites (*ρ* = −0.16, *p* = 0.68).

### 3.4 Relatedness Among Samples and MLLs

Mean pairwise Wang's *r* among all samples was 0.041 (SD = 0.42) and among only unique MLLs at each site was −0.04 (SD = 0.33). When tidal regimes were analyzed separately, permutation tests indicated that all nontidal samples ($\bar{x}$ = 0.33, SD = 0.47) and MLLs ($\bar{x}$ = 0.25, SD = 0.31) were substantially more closely related to each other than expected, whereas tidal samples ($\bar{x}$ = −0.13; SD = 0.28) and MLLs ($\bar{x}$ = −0.08 with SD = 0.27) were less related than expected (all *p* < 0.0001). These differences yielded higher relatedness in the nontidal than the tidal regime when considering all samples and only unique MLLs (both with *p* < 0.0001). Mean relatedness between the tidal regimes was *−*0.145 (SD = 0.25) for all samples and *−*0.117 (SD = 0.25) for all MLLs.

**FIGURE 5:**
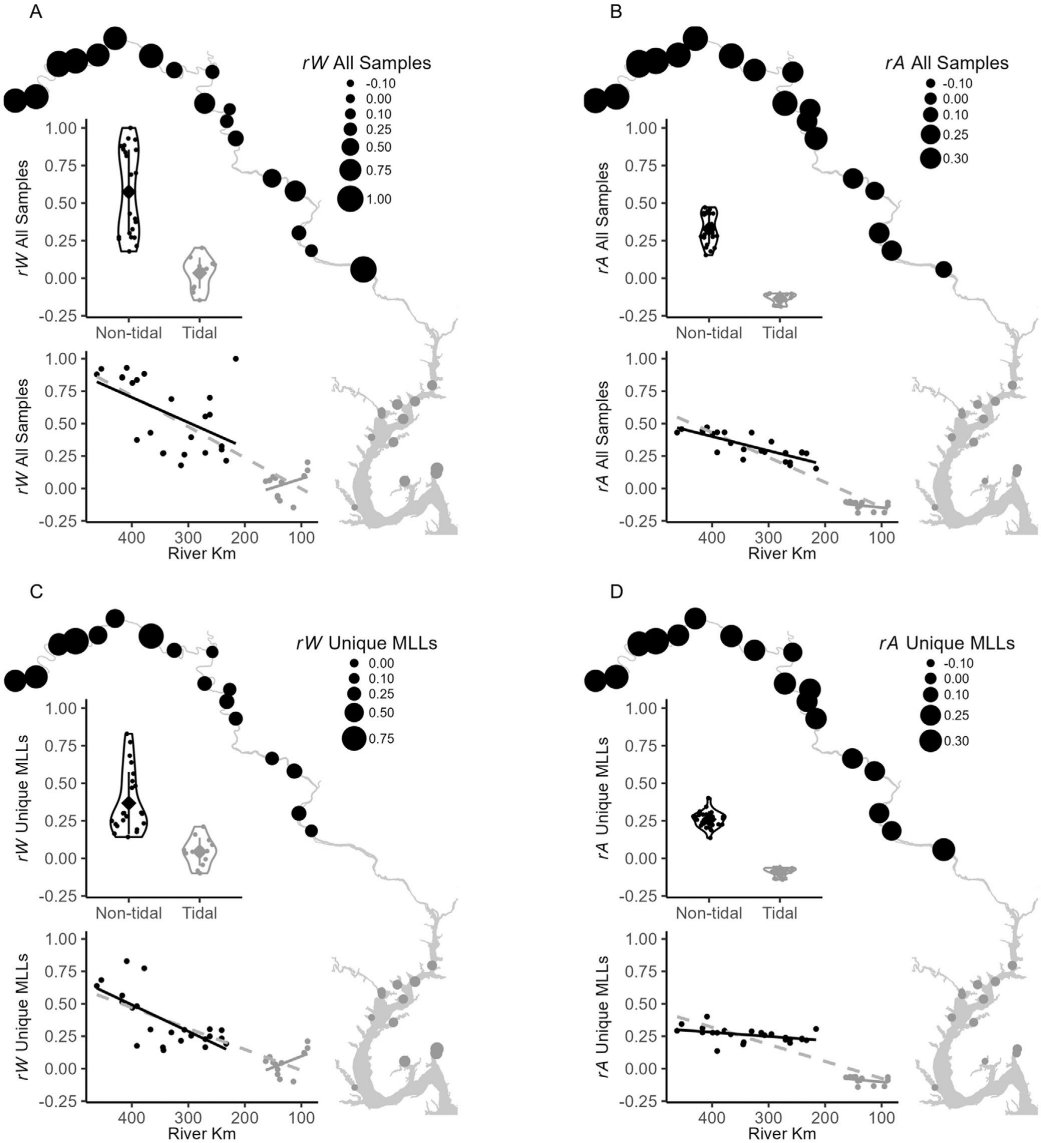
Wang's pairwise relatedness among all samples (A, B) and among unique multilocus lineages (MLLs; C, D) within the same site (*r*_*W*_; A, C) and at different sites (*r*_*A*_; B, D) in tidal (gray) and nontidal (black) portions of the river. Nontidal and tidal reaches are compared statistically using violin plots and correlations of relatedness with river kilometer within each tidal reach (solid lines) and across the entire river (gray dashed line). Mean values among samples are indicated by the gray diamonds and error bars are one standard deviation. *Y*‐axis extents are standardized to allow comparison across statistics.

Pairwise comparisons of all samples from the same site yielded average *r*_*W*_ of 0.37 (SD = 0.39) and those from different sites in the same tidal regime averaged *r*_*A*_ of 0.15 (SD = 0.27). When considering only unique MLLs, *r*_*W*_ averaged 0.28 (SD = 0.26) and *r*_*A*_ averaged 0.15 (SD = 0.17). Relatedness among all nontidal samples (Figure 5A,B) was substantial whether they came from the same site (*r*_*W*_: $\bar{x}$ = 0.59, SD = 0.32) or from different (*r*_*A*_: $\bar{x}$ = 0.32, SD = 0.16; Figure 5) sites. By contrast, samples from tidal sites indicated no deviation from expected relatedness within sites (*r*_*W*_: $\bar{x}$ = 0.018, SD = 0.16) and lower relatedness than expected among (*r*_*A*_: $\bar{x}$ = −0.14; SD = 0.112; Figure 5A,B) sites. When only unique MLLs were examined (Figure 5C,D), relatedness in nontidal sites remained high both within (*r*_*W*_: $\bar{x}$ = 0.39; SD = 0.24) and among sites (*r*_*A*_: $\bar{x}$ = 0.26; SD = 0.06). Relatedness among MLLs from tidal sites remained low whether they were from the same (*r*_*W*_: $\bar{x}$ = 0.04; SD = 0.09) or different sites (*r*_*A*_: $\bar{x}$ = −0.09; SD = 0.03). All observed differences in *r*_*W*_ and *r*_*A*_ between tidal and nontidal reaches were greater than all 10,000 permutations (Figure 5).

Pairwise relatedness in all the 36 sites yielded a positive correlation for both *r*_*W*_ and *r*_*A*_ with river km (Figure 5) when all samples were included (*r*_*W*_: *ρ* = 0.79; *p* = 9.1*e* ^−9^; *r*_*A*_: *ρ* = 0.92; *p* = 8.8*e* ^−16^) and when only unique MLGs at each site were included (*r*_*W*_: *ρ* = 0.75; *p* = 1.67*e* ^−7^; *r*_*A*_: *ρ* = 0.83; *p* = 3.21*e* ^−10^). Relatedness based on all samples (Figure 5A,B) was positively correlated with river km in nontidal reaches (*r*_*W*_ *ρ* = 0.47; *p* = 0.02 and *r*_*A*_ *ρ* = 0.82; *p* = 9.6*e* ^−6^), but not in the tidal reaches (*r*_*W*_ *ρ* = −0.27; *p* = 0.40; *r*_*A*_ *ρ* = 0.4; *p* = 0.20). The same relationships held when considering only unique MLLs (nontidal *r*_*W*_ *ρ* = 0.41; *p* = 0.046 and *r*_*A*_ *ρ* = 0.50; *p* = 0.013; and tidal *r*_*W*_ *ρ* = *−*0.24; *p* = 0.46 and *r*_*A*_ *ρ* = 0.46; *p* = 0.13; Figure 5C,D). Thus, all samples and unique MLLs from both the same and different sites became more closely related to each other as one moved upstream only in the nontidal regime. Higher relatedness in the nontidal regime drove the positive correlation at the river scale.

### 3.5 Genetic Diversity

**FIGURE 6:**
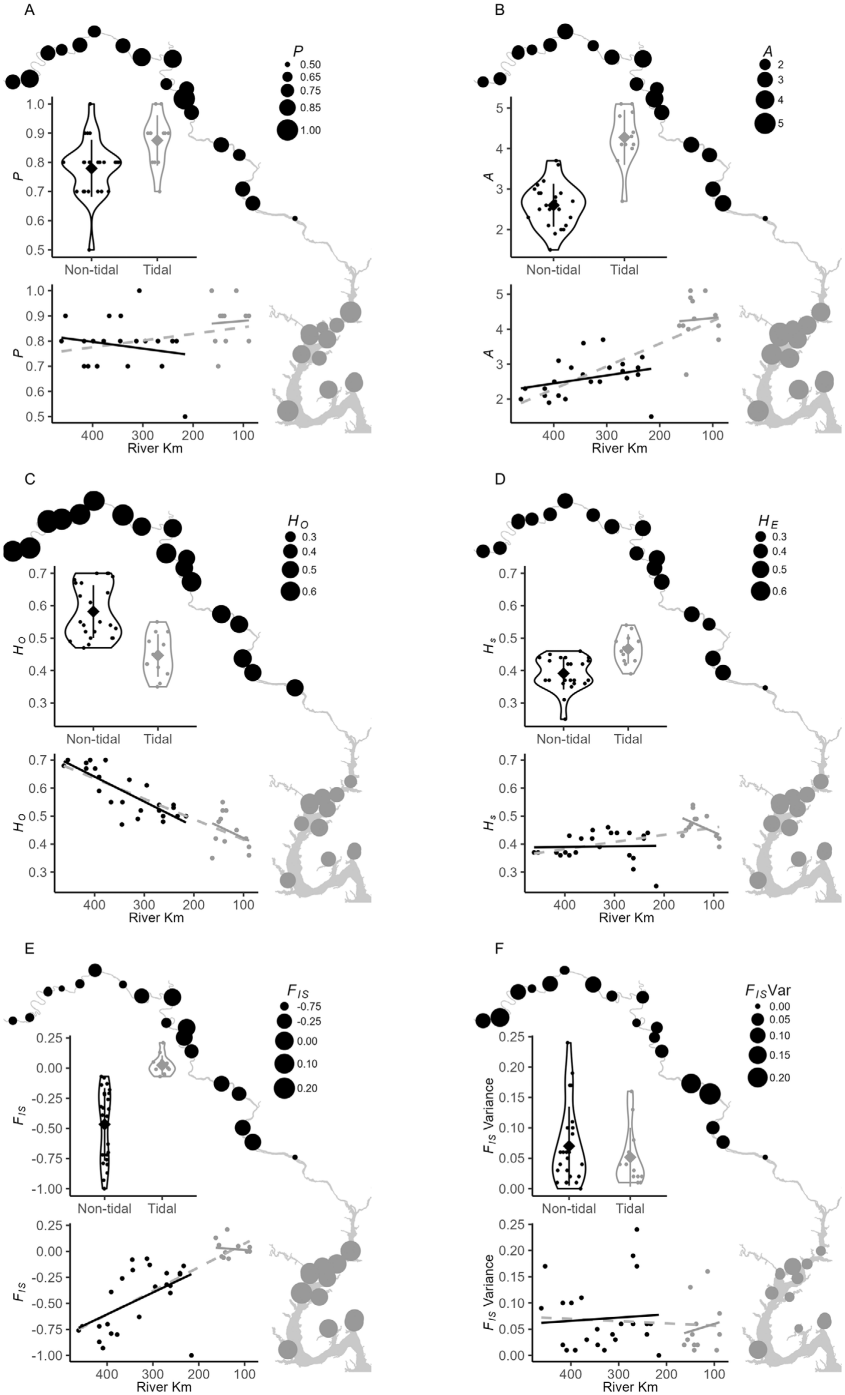
Within‐site genetic diversity statistics (A) *P*, (B) *A*, (C) *H*_*o*_, (D) *H*_*s*_, (E) mean *F*_IS_ across loci, and (F) variance in *F*_IS_ across loci in tidal (gray) and nontidal (black) portions of the river. Nontidal and tidal reaches are compared statistically using violin plots and correlations with river km within each tidal reach (solid lines) and across the entire river (gray dashed line). Values for each site are means of pairwise differences of that site to all other sites in the same tidal regime. *Y*‐axis extents are standardized to allow comparison across statistics.

We detected 79 alleles across 10 microsatellite loci (range = 5–13 per locus). The nontidal regime supported 57 alleles and the tidal supported 70; 48 alleles were common to both regimes. Although the loci were polymorphic overall and within each tidal regime, within‐site *P* averaged 0.88 (SD= 0.10). Nontidal sites were less polymorphic ($\bar{x}$ = 0.78; SD = 0.10) than tidal sites ($\bar{x}$ = 0.87; SD = 0.09; *p* = 9.7 *e* ^−3^; Figure 6A).

When considering all samples across both tidal regimes, within‐site *A* averaged 3.16 (SD = 0.98). Considered separately, *A* was lower in nontidal sites ($\bar{x}$ = 2.60; SD = 0.53) than tidal sites ($\bar{x}$ = 4.27; SD = 0.67; *p* = 0.0001; Figure 6B). These differences remained significant when alleles were rarefied (*p* = 0.0001). Strong negative correlation between *A* and river km (*ρ* = *−*0.77, *p* = 3.9*e* ^−8^) was driven by the difference between nontidal and tidal sites. The correlation was weak in the nontidal region (*ρ* = *−*0.43, *p* = 0.035) and absent for the tidal region of the river (*ρ* = *−*0.17, *p* = 0.59). We also saw a negative correlation for *A*_*r*_ when considering all sites (*ρ* = *−*0.77, *p* = 3.2*e* ^−8^), a weak relationship for nontidal sites (*ρ* = *−*0.41, *p* = 0.05), but no relationship (*ρ* = 0.032, *p* = 0.92) across tidal sites.

Average *H*_*o*_ within all sample sites was 0.54 (SD= 0.10), and was higher in nontidal ($\bar{x}$ = 0.58, SD = 0.08) than tidal sites ($\bar{x}$ = 0.45, SD = 0.06; *p* = 0.0001; Figure 6C). We found a strong correlation between site‐level *H*_*o*_ and river km (*ρ* = 0.81, *p* = 2.1*e* ^−9^), indicating lower *H*_*o*_ in downstream locations. The relationship remained significant in the nontidal regime (*ρ* = 0.77, *p* = 9.3*e* ^−6^), but not the tidal (*ρ* = 0.17, *p* = 0.60). *H*_*s*_ averaged 0.42 (SD = 0.06) across all sample sites, and was lower in nontidal ($\bar{x}$ = 0.39, SD = 0.05) than tidal sites ($\bar{x}$ = 0.47, SD = 0.05; *p* = 0.0001; Figure 6D). We found a strong correlation between site‐level *H*_*s*_ and river km (*ρ* = −0.54, *p* = 7.5*e* ^4^) at the scale of the river, but not in the nontidal (*ρ* = −0.13, *p* = 0.54) or tidal (*ρ* = 0.24, *p* = 0.45). Thus, lower *H*_*s*_ in downstream locations was due to differences between tidal regimes.

Site‐level *F*_IS_ values averaged *−*0.30 overall (SD = 0.34) and indicated heterozygote deficit in 2 tidal sites and heterozygote excess in 21 nontidal sites. Mean *F*_IS_ values (Figure 6E) for nontidal ($\bar{x}$ = −0.47; SD = 0.30) and tidal ($\bar{x}$ = 0.02; SD = 0.08) sites reflected those differences (*p <* 0.0001; Figure 6E). Overall negative correlation between *F*_IS_ and river km indicated increasing heterozygote excess when moving upstream through the entire river (*ρ* = *−*0.80, *p* = 4.01*e* ^−9^). This pattern was driven by the observed negative correlation in the nontidal (*ρ* = *−*0.47, *p* = 0.021) as the tidal showed no correlation between *F*_IS_ and river km (*ρ* = 0.03, *p* = 0.92). Variance in *F*_IS_ across loci within sites (Figure 6F) was similar across the river (*p* = 0.411), averaging 0.06 (SD = 0.06) overall, 0.07 (SD = 0.07) in nontidal sites, and 0.05 (SD = 0.05) in tidal sites, with no correlation with river km at any scale (all *p* ≥ 0.64).

**FIGURE 7:**
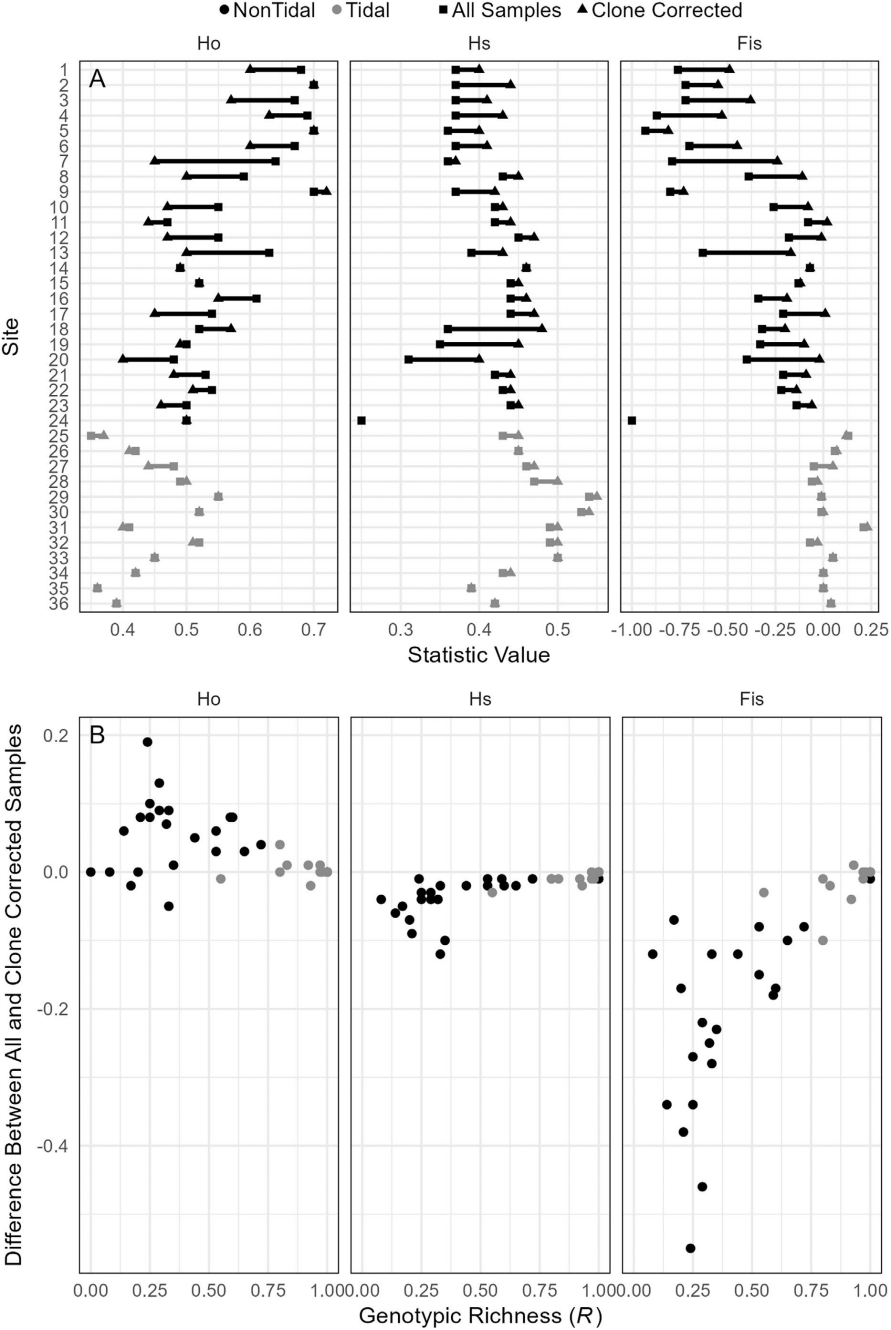
(A) Comparison of *H*_*o*_, *H*_*s*_, and mean *F*_IS_ calculated using all samples (triangles) and after clone correction (i.e., one sample of each MLL at each site; squares). The deviation between values at each sampled site (black lines and symbols are nontidal sites and gray tidal sites) highlight the extent to which each measure is affected by clonality. (B) Association between deviations and genotypic richness (*R*).

Clone‐correction substantially altered *H*_*o*_, *H*_*s*_, and *F*_IS_ values in sites that supported clonal replicates (Figure 7). Although the direction of deviation in *H*_*o*_ varied, it was typically lower in clone‐corrected samples. By contrast, clone‐corrected *H*_*s*_ was always lower when any clones were present, and this difference yielded substantially lower *F*_IS_ values. Deviations were obvious even at *R* values of 0.9, and the lowest *H*_*s*_ and *F*_IS_ values were associated with *R* ranging from ~0.2–0.4 (Figure 7B).

### 3.6 Genetic Structure Among Sites and Tidal Regions

Among all sites, overall *F*_ST_ was 0.155, $G_{\mathrm{ST}}^{\prime \prime }$ was 0.243, and *D*_est_ was 0.119. Global values among nontidal sites (*F*_ST_: 0.128, $G_{\mathrm{ST}}^{\prime \prime }$: 0.175, and *D*_est_: 0.077) were similar to those among tidal sites (*F*_ST_: 0.092, $G_{\mathrm{ST}}^{\prime \prime }$: 0.175, and *D*_est_: 0.090). Global values for tidal versus nontidal samples were (*F*_ST_: 0.099, $G_{\mathrm{ST}}^{\prime \prime }$: 0.187, and *D*_est_: 0.10), indicating similar low gene flow between the tidal regimes and among sites within each region. Confidence intervals for all global estimates of the three measures indicated values were greater than 0.

**FIGURE 8:**
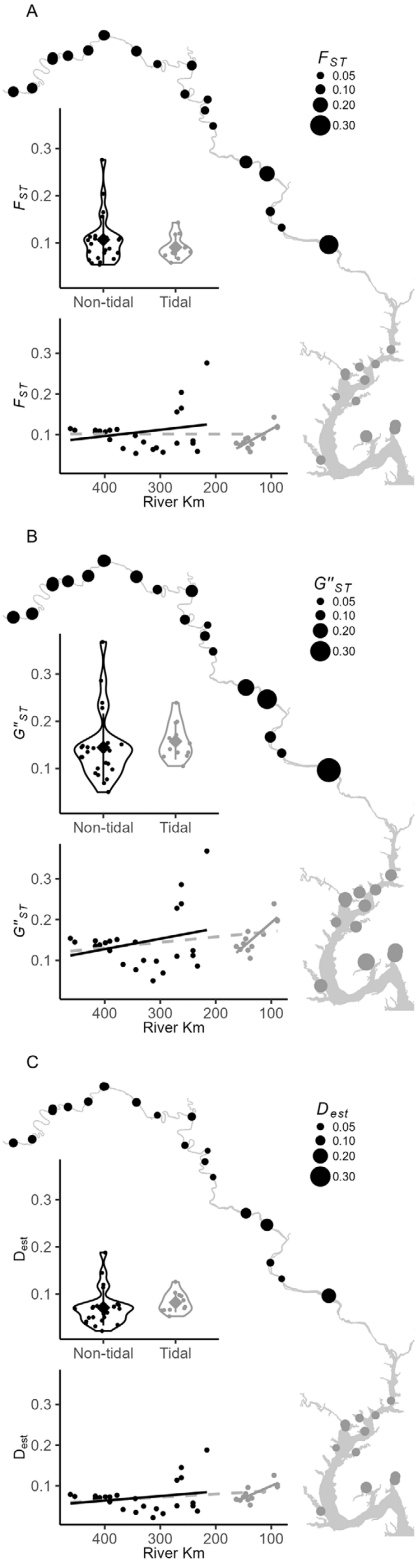
Among‐site genetic diversity statistics (A) *F*_ST_, (B) $G_{\mathrm{ST}}^{\prime \prime }$, and (C) *D*_est_ by site and tidal regime in tidal (gray) and nontidal (black) portions of the river. Nontidal and tidal reaches are compared statistically using violin plots and correlations with river km within each tidal reach (solid lines) and across the entire river (gray dashed line). Mean values among samples are indicated by the gray diamonds and error bars are one standard deviation.

Mean values from pairwise comparisons of each site to all other sites were *F*_ST_ (0.139), $G_{\mathrm{ST}}^{\prime \prime }$ (0.219), and *D*_est_ (0.114). Values of all three measures from pairwise comparisons among nontidal sites (*F*_ST_ = 0.091, $G_{\mathrm{ST}}^{\prime \prime }$ = 0.157, and *D*_est_ = 0.082) were not different from comparisons among nontidal sites (*F*_ST_ = 0.107, $G_{\mathrm{ST}}^{\prime \prime }$ = 0.199, and *D*_est_ = 0.102; Figure 8A–C) with permutation tests yielding *p*‐values of 0.34, 0.57, and 0.37, respectively.

**FIGURE 9:**
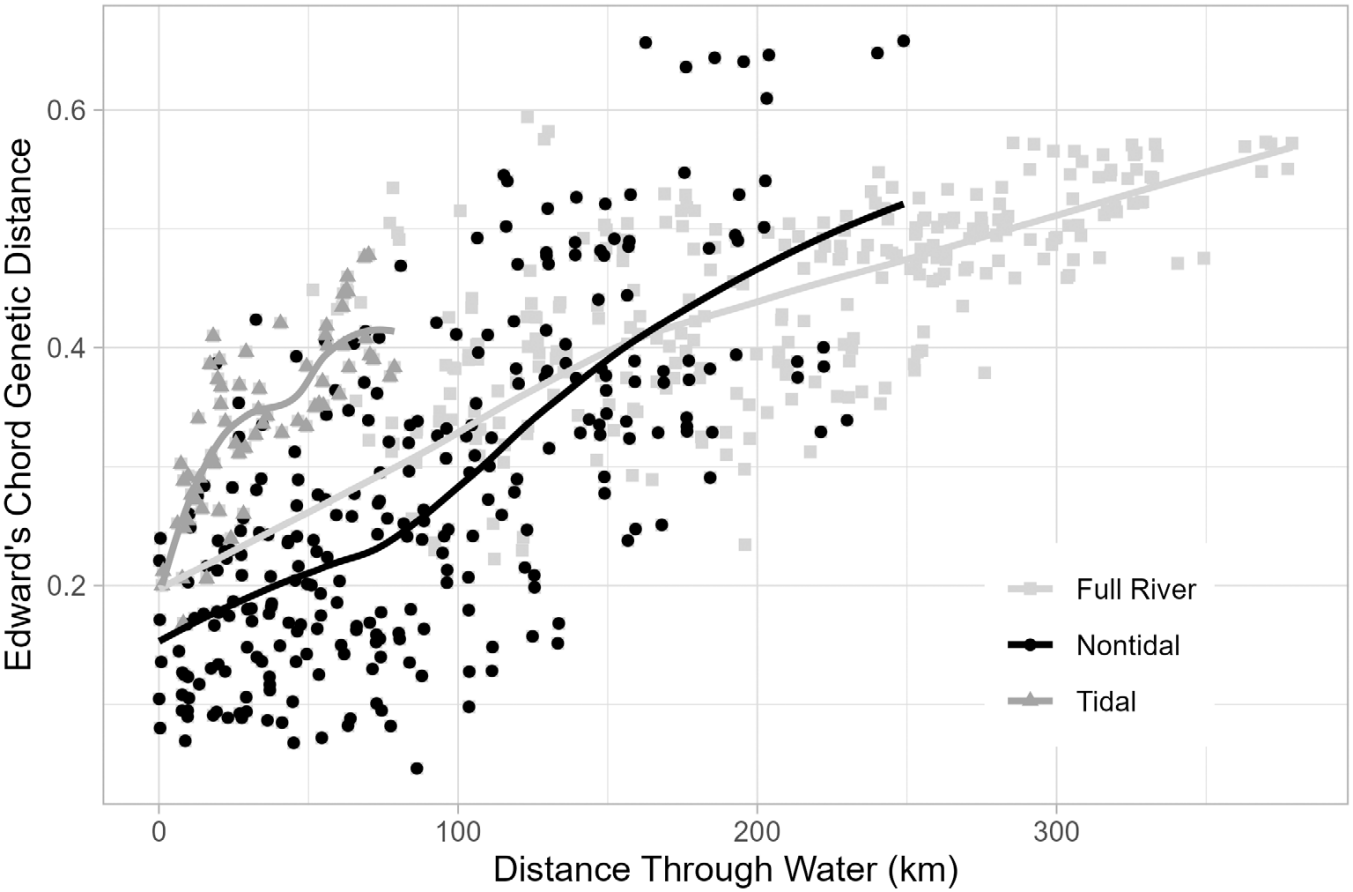
Scatterplot of Edward's chord genetic distance by geographic distance through water for the full river, nontidal sites, and tidal sites.

IBD based on correlation of Edward's chord genetic distance and geographic distance among sites through water was strongly positive for all samples from all sites combined (Mantel's *r* = 0.72), from only nontidal sites (Mantel's *r* = 0.70), and from only tidal sites (Mantel's *r* = 0.77), all with *p* = 0.001 (Figure 9). Although all three Mantel's r values were similar, the relationships were nonlinear and differed in the spatial scale of inflections in the slope. Tidal sites increased most rapidly up to ~30 km and again between 50 and 75 km (Figure 9). Increases in distances among nontidal sites consisted of two relatively linear domains, with a shallower slope among sites within ~80 km of one another. When all sites were considered, two linear domains were observed, with a slightly shallower slope for distances among sites that were greater than ~160 km apart (including both nontidal sites and sites between tidal regimes).

**FIGURE 10:**
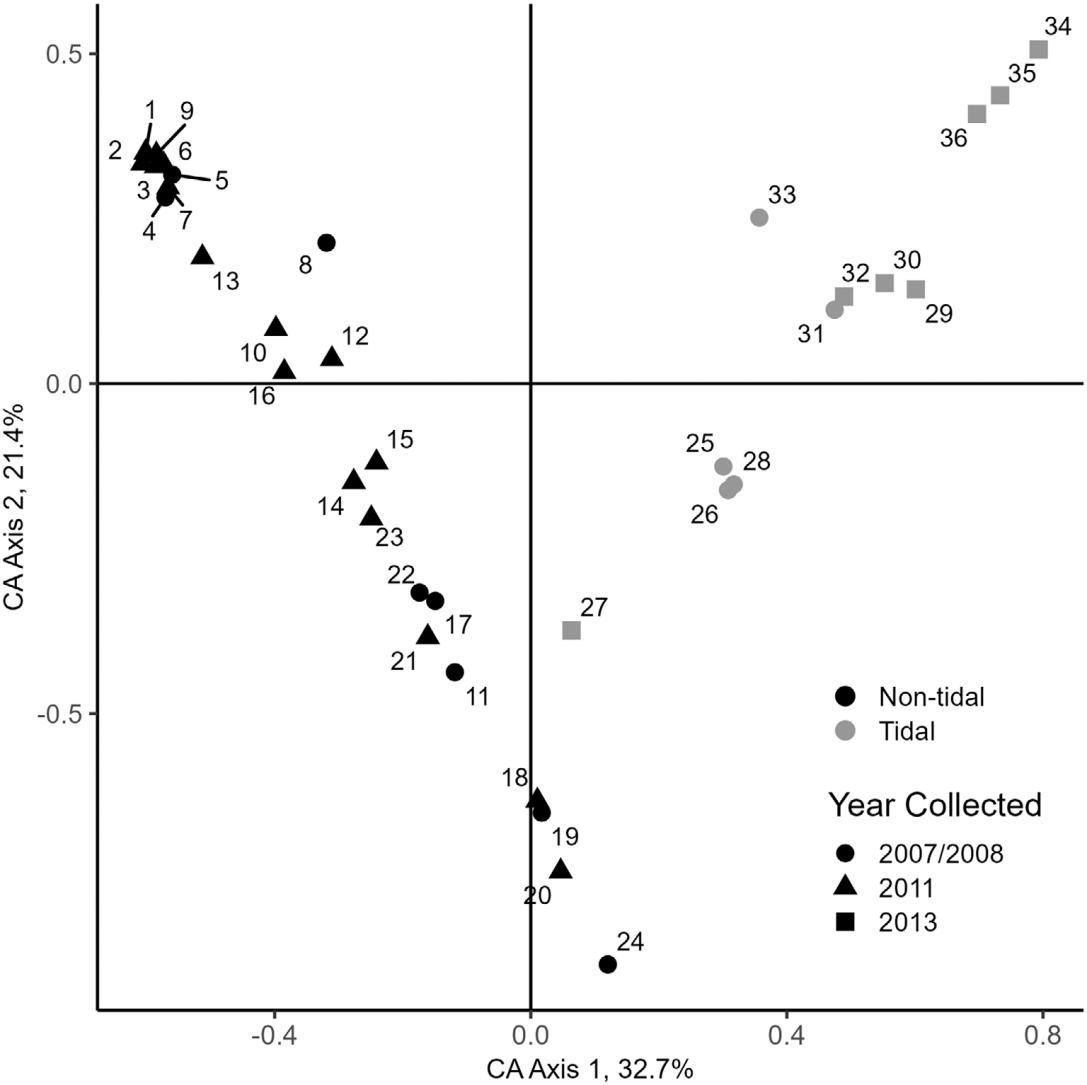
First and second axes of a correspondence analysis (CA) based on allele frequencies of all samples at 36 sites. Sites from 2007 to 2008 are from Lloyd et al. (2011). The interspersion of sites from different years indicates no large changes in allele frequencies across time.

In the correspondence analysis, Axis 1 explained 32.7% of the variation and Axis 2 explained 21.4%. Axis 1 mostly separated tidal from nontidal sites (Figure 10). Nontidal sites were arrayed in relatively even intervals from upstream to downstream along increasing values of Axis 1 and decreasing values of Axis 2. Similarity in CA scores was more related to geographic proximity than the time period of sampling. Even so, geographically proximate sites were not always closest in ordination space (Figure 10). Tidal sites occupied a larger extent of both axes, with larger and more uneven ordination distances among sites (Figure 10). Arrangement of tidal sites also generally reflected location along the river gradient, with more downstream locations having higher values on Axis 2. Sites from different time periods were intermixed along the CA gradients. Site 27 is anomalous among tidal sites in that it appeared more similar to nontidal sites on Axis 1. This site supported only three MLLs and their allele frequency profiles differ from those of other tidal sites.

## 4 Discussion

Examining the spatial genetic diversity in *V. americana* across its range in the riverine and estuarine Potomac River gave insight into the relative effects of continuous hydrologic connectivity versus tidal regime on genetic structure. If hydrologic connectivity is the dominant factor throughout the river, we expect weak overall genetic structure, relatively constant declines in similarity with distance, and downstream accumulation of genetic and genotypic diversity. If tidal regime is a stronger force, we expect more idiosyncratic spatial genetic structure among populations in the bidirectional and localized currents of the Potomac River's tidal regime than in the nontidal. We did find evidence for continuous hydrologic connectivity in the entirety of the river in terms of significant IBD (Figure 9) and lower diversity upstream for most, but not all, diversity measures (Figures 3 and 6). However, lower downstream diversity was mostly associated with the dramatic disjunction in levels and structure of genotypic diversity coinciding with the transition from nontidal to tidal environments (Figures 3 and 6). The amount of variation partitioned between the two environments (*F*_ST_ = 0.099) fell between levels documented from hydrologically connected versus disconnected aquatic systems. For example, *V. spinulosa* collected from sites along the Yangtze River in China had *F*_ST_ = 0.06 (Chen et al. 2007), whereas *F*_ST_ in *V. natans* and *V. spinulosa* from hydrologically isolated lakes along the same river was 0.132 and 0.202, respectively (B. Wang et al. 2010). In *V. americana* , *F*_ST_ between the Potomac and Chesapeake Bay was 0.114 (Lloyd et al. 2011).

When tidal sites were considered separately, downstream accumulation of diversity was seen only for *R* (Figure 3A), and even that was only marginally significant (*p* = 0.048). Stronger and more nonlinear IBD (Figure 9) and greater and more irregular CA distance among tidal sites (Figure 10) indicate the relatively chaotic patterns that are typical of marine environments (Becheler et al. 2010; Jahnke et al. 2018; Johnson and Black 1984; Selkoe et al. 2010; Siegel et al. 2008; Sinclair, Gecan, et al. 2014). In contrast, the relatively linear IBD and continuous distribution variation in CA ordination space in the nontidal environment were consistent with riverine hydrologic connectivity. However, we saw downstream accumulation only for *A* (Figure 6B), whereas *H*_*o*_ was higher in more upstream locations (Figure 6C). Thus, rather than seeing a continuous increase along the river, the fall line marks an abrupt change in structure and diversity, indicating that tidal influence disrupts hydrologic connectivity. Below, we discuss how the interplay between local environmental conditions and species biology affects genotypic and genetic structure and yields different outlooks for resilience for the same species growing in different parts of the same river.

### 4.1 Shifting Life‐History Patterns Across Tidal Regimes

Observed differences in genetic structure between nontidal and tidal environments reflect shifting reproductive modes that drive MLL diversity and dominance (Figure 3), and relatedness among those MLLs (Figure 5) in the two environments. A broad range of genotypic diversity is common across the ranges of long‐lived, outcrossing, partially clonal aquatic species (Becheler et al. 2010; Procaccini et al. 2001; Sinclair et al. 2020), including *V. americana* (Marsden et al. 2022; Ngeve et al. 2023). Even so, the fundamental shift from predominantly asexual reproduction throughout the nontidal reaches to almost exclusively sexual reproduction in tidal sites in the same river (Figure 3) was extraordinary. Given this, it is worth exploring the support for the accuracy of our assessment of clonality. First, based on modeling by Stoeckel et al. (2021), our sample sizes at each site likely underestimate true rates of clonality, but we have no reason to expect that underestimates would differ between the tidal environments. Second, genetic methods can underestimate clonality if MLG assignments are based on somatic mutations or genotyping errors, and can overestimate it if too few loci with sufficient allelic diversity are sampled. We guarded against underestimates by collapsing very similar MLGs into MLLs and confirming those MLLs have an extremely low probability of occurring by chance if they were not from the same zygote. The risk of overestimating clonality is present but low. The genotype accumulation curve for the dataset (Figure 2) shows all MLLs are distinguished by seven loci, and that all 10 loci are necessary to distinguish all MLGs. There were very small incremental increases in MLGs after 7 loci, but the 1% increase at the final step from 9 to 10 loci indicates that sampling more loci would likely distinguish additional MLGs. Because allelic richness was lower in the nontidal, it is challenging to distinguish whether low allelic diversity limited detection of unique MLLs, or if it was a consequence of high rates of clonal reproduction and extreme dominance of a few MLLs. With low allelic diversity, sexually reproducing populations are expected to have high homozygosity, whereas highly clonal populations are expected to have high heterozygosity (Meirmans 2024). The higher *H*_*o*_ (Figure 6C) and lower *F*_IS_ (Figure 6E), despite extremely high levels of relatedness (Figure 5), were consistent with high rates of clonal reproduction. Moreover, Meirmans (2024) has suggested that clone‐corrected *H*_*o*_ and F_IS_ represent effects of sexual reproduction, whereas estimates calculated using all samples include effects of both sexual and clonal reproduction. The differences we observed in these values (Figure 7) strongly support high levels of clonality in nontidal sites. Pareto *β* has also been suggested to distinguish between clonal and sexual populations (Stoeckel et al. 2021) and indeed we found lower values in nontidal sites (Figure 3D). However, Pareto *β* was a less informative measure because it is undefined when every sample is the same MLL and when every sample is unique, and therefore does not capture the full clonal–sexual reproduction gradient. Given these multiple lines of evidence, we are confident in our conclusion that the reproductive mode differs between the two tidal regimes. It is unlikely that additional MLGs from more loci or additional replicates of existing MLGs from more samples would fundamentally change this inference (Meirmans 2024).

Beyond simply the amount of clonal reproduction, the spatial extent of multiple nontidal MLLs indicates a magnitude of vegetative dispersal (Figure 4) that exceeds reports for other submersed aquatic plants. For example, 12 MLLs were larger than *Zostera noltei* clones (24 km; Berković et al. 2018), and eight of those were larger than a *Thalassia testudinum* “megaclone” (47 km; Bricker et al. 2018). Three had greater extents than a *Posidonia australis* clone (180 km; Edgeloe et al. 2022), considered to be the world's largest plant. The above species are marine seagrasses that form meadows and are dispersed by ocean currents and waves. Seven of our 12 widespread MLLs extended at least 104 km along the river (Figure 4), yielding multiple clones that are among the most extensive known. As noted by Lloyd et al. (2011), these extents likely involved dispersal of vegetative propagules rather than incremental stoloniferous growth. The high apparent dispersal capability of vegetative propagules indicates high connectivity over the 246 km extent of the species in the nontidal Potomac (Figure 4). Thus, the complete lack of those MLLs in tidal reaches is surprising and indicates the fall line represents a strong barrier to vegetative dispersal. Whether that barrier is the fall line itself or differing environmental conditions on either side of it is uncertain.

Given the high degree of clonality and the large extent of multiple MLLs, the high relatedness among all sampled shoots in the nontidal (Figure 5A,B) is not surprising given clonal shoots have *r* = 1. What was surprising was the high relatedness among different nontidal MLLs (Figure 5C,D), even ones that were not growing at the same site. This high degree of relatedness across such a large spatial scale could depress the potential for adaptation beyond what the extremely low number of nontidal MLLs already suggests. This is in contrast to the tidal reaches in which samples and MLLs within sites are not more related than expected and are less related than expected across sites (Figure 5).

### 4.2 Potential Drivers of Differentiation and Variation in Genetic Structure

We explore three possible explanations for high apparent clonal reproduction in nontidal reaches when it is so rare in tidal reaches: (1) differing mechanisms of recruitment and persistence, (2) limited opportunity for pollination in directional water flow, and (3) relative stability. Although we offer these explanations, we recognize that the differences cannot be attributed exclusively to tidal regime because we have found similarly low *R* in tidal sites in the Hudson River of New York (Marsden et al. 2022; Ngeve et al. 2023).

Genotypic diversity in submersed aquatic plants is influenced by bed establishment and continued recruitment. Two strategies that yield different levels of genotypic diversity have been proposed (Silvertown 2008). In one, beds establish through initial seedling recruitment (ISR; Eriksson 1993) and further develop via asexual expansion of the initial recruits (Silvertown 2008). Under ISR, initial diversity is a function of the seed abundance and their allelic diversity and relatedness. Diversity will decrease as beds age because more shoots will be clonal replicates. Increased unevenness due to different rates of clonal reproduction across the colonists will further decrease diversity (Chen et al. 2007; Piquot et al. 1998). The alternative strategy, repeated seedling recruitment (RSR; Alberto et al. 2005; Eriksson 1993), yields more genotypic variation through ongoing sexual reproduction. Genetic diversity and relatedness will depend on whether continued recruitment is from matings among initial recruits or includes new immigrants. The ISR strategy is thought to be common in stable environments and the RSR strategy is associated with moderate disturbance (Becheler et al. 2014; Eriksson 1993).

Tidal Potomac sites conform to the RSR strategy in that almost all samples resulted from sexual reproduction (Figure 3). The small amount of asexual reproduction was localized to a median extent of < 24 m, and the largest extent was 1.5 km in the same bay (in Sites 29 and 30). Low relatedness among sampled shoots within sites (Figure 5) indicates large populations or ongoing recruitment from other sites. However, significant inbreeding coefficients at three tidal sites (25, 31, and 33) could indicate mating among initial recruits and their offspring, but it is curious this is not reflected in relatedness. Despite being statistically significant, the levels of inbreeding were relatively low and could also be due to Wahlund effects if the intrasample distances exceeded local breeding neighborhoods (Neel et al. 2013; Waples 2015).

Nontidal sites conformed to neither ISR nor RSR in that the extent of vegetative propagules cannot be explained by localized incremental stoloniferous growth. Lloyd et al. (2011) noted the time needed for observed expansion of their two largest *V. americana* MLLs (160 and 132 km) exceeded the time that habitats have been in place in the Potomac. We have documented even larger extents here as well as large extents of additional MLLs. Clones of *Posidonia oceanica* that are an order of magnitude smaller than the ones we have found (up to 15 km) have been considered some of the oldest organisms on the earth (Arnaud‐Haond et al. 2012). We suggest that the extensive clones in the nontidal Potomac result from a third strategy—repeated vegetative recruitment (RVR)—in which populations are frequently initiated by dispersing vegetative propagules and perpetuated by local vegetative extension and repeated vegetative dispersal. Evidence for hydrochory of asexual propagules is emerging in a number of seagrasses (Berković et al. 2018; Lai et al. 2018), and we have observed vegetative propagules of *V. americana* grown under greenhouse conditions dislodging from the sediments in the spring when leaves emerge but roots are still short. The plants can float for weeks while growing longer roots that enable establishment in new locations.

As individual MLLs of this dioecious species become dominant over large spatial scales, eco‐evolutionary feedbacks emerge because plants of the opposite sex can become isolated beyond effective pollination distances, which are on the order of a few meters (Lloyd et al. 2018). Nontidal environments may also have more limited opportunities for pollination given that rapid, directional currents carry staminate flowers out of beds and away from pistillate flowers (Sullivan and Titus 1996) and hold pistillate flowers below the water surface where pollination occurs. Low levels of pollination will favor vegetative expansion, leading to even larger extents of individual MLLs, and even further limiting sexual reproduction (Barrett 2015; Honnay and Bossuyt 2005; Lokker et al. 1997, 1994; Lovett‐Doust and Laporte 1991). Sites dominated by one or a few MLLs then provide vegetative propagule pressure that fuels downstream dispersal and amplifies the clonal extent and dominance across multiple sites. If most shoots at a site are clonal replicates, the only natural source of new genotypic diversity is dispersal from other sites, which is most often accomplished through water. The potential for dispersal to be a source of new diversity depends on proximity to and diversity in established populations (McMahon et al. 2014). Given the low diversity, extensive clonality, and high relatedness among MLLs in the 246 km extent of the species in the nontidal, there is no proximal source of increased diversity. The potential for new diversity is limited to very low probability long‐distance dispersal events. The strong disjunction between the tidal and nontidal regions indicates such events have not been common.

The contrasting lack of extensive clonality in the tidal reaches indicates sufficient pollination for sexual reproduction or lower rates of asexual growth. Pollination is potentially facilitated by tidal flow that can slow or reverse downstream currents and retain male flowers in *V. americana* beds longer. Further, French and Moore (2003) have shown that fewer ramets are produced at higher experimental salinity levels, but variation among treatments in turion production provides only suggestions of reduced asexual reproduction at higher salinity sites. Although diversity is higher within tidal sites, differentiation among them and lack of shared clonal diversity indicate that distances are too large for regular dispersal to be a source of that diversity. Dispersal is expected to be especially limited in the more downstream locations where suitable habitat is confined to lower salinity coves or tributaries that are separated by the deeper, wider, and more saline main channel.

Differences in the relative environmental stability also potentially drive differences in tidal versus nontidal genotypic structure (Banks et al. 2013; McMahon et al. 2017). As with all rivers, the whole Potomac is dynamic and both reaches are exposed to periodic disturbances. However, directional freshwater flows in the nontidal create relatively consistent environmental conditions compared to the semi‐diurnal and seasonal fluctuations in salinity, temperature, and turbidity in tidal sites. Relatively stable environments favor expansion via asexual reproduction, whereas more dynamic, disturbance‐prone environments may limit it (Cao et al. 2021; Sinclair, Krauss, et al. 2014). For example, open‐water meadows of the seagrass *Posidonia australis* with moderate levels of disturbance had higher levels of genotypic and allelic diversity than highly exposed sites (Sinclair, Krauss, et al. 2014). This relationship is not ubiquitous, as the freshwater species *Potamogeton pectinatus* had higher clonal diversity in pond habitats than in adjacent more dynamic river sites (Triest and Fenart 2014) and sites with extreme disturbance may favor clonality (Alberte et al. 1994; Micheli et al. 2005). The changing environmental conditions in tidal sites may also alter dynamics with other species in ways that limit extensive vegetative growth of *V. americana* . For example, annual variation in salinity, turbidity, and temperature favors the invasive species *Hydrilla verticillata* and reduces *Vallisneria* cover and stem numbers some years (Carter and Rybicki 1986; Rybicki et al. 2001).

### 4.3 Implications for Resilience

The major threats to persistence of *V. americana* in the Potomac remains chronic sedimentation and eutrophication that was in place by the 1930s (Haramis and Carter 1983; Rybicki et al. 2001), combined with periodic major storm impacts (Orth et al. 2017). Natural resurgence between 1983 and 1993 (Carter and Rybicki 1986; Rybicki et al. 2001) and stability through 2005 (Karrh et al. 2007; Rybicki et al. 2007) was associated with favorable flow and weather conditions that increased water clarity. Increases in submersed aquatic plants associated with nutrient reductions are seen more broadly in the Chesapeake Bay as well (Lefcheck et al. 2018; Orth et al. 2017). The importance of improved water clarity to bed growth and persistence is clear, even if it does not yield complete ecosystem recovery (sensu Duarte et al. 2013). Relatively high genotypic and allelic diversity in tidal sites indicates no immediate concern that limited genetic diversity might compromise long‐term resilience beyond the ecological threats. Controlled experiments testing for capacity to adapt or acclimate to temperatures and salinity levels anticipated under climate change are necessary to fully understand risk given changes to species abundances already observed (Richardson et al. 2018).

The perceived risks and benefits of extensive clonal growth as observed in the nontidal Potomac are mixed (Eckert et al. 2016; Vallejo‐Marín et al. 2010). Some authors consider highly clonal populations an evolutionary dead end (Honnay and Bossuyt 2005) given that without genetic variation on which natural selection can act, long‐term prospects for adaptation will be dim. Although somatic mutations will likely have accumulated (Reusch and Bostrom 2011; Yu et al. 2020), the probability that those mutations would be adaptive is extremely low, leaving acclimation through phenotypic plasticity as the only means of responding to environmental change (Pazzaglia et al. 2021). In this regard, epigenetic variation, as in Jueterbock et al. (2020), could be vital to future persistence. Other research emphasizes how clonal growth can enable immediate resilience by facilitating persistence through and recovery from disturbance in conditions that do not favor sexual reproduction (de Witte and Stocklin 2010; Fahrig et al. 1994; Ngeve et al. 2023; van der Merwe et al. 2010). Large clones have even been suggested as a key strategy to surviving changing climate (Bricker et al. 2018).

Given these insights into the risks and benefits of clonal growth, one can now begin to explore whether the high heterozygosity that emerges as a consequence of clonality (Meirmans 2024; Stoeckel et al. 2021) confers fitness or acclimation potential to clonal individuals. Indeed, higher fitness in outcrossed than inbred individuals is one of the most fundamental observations in plant evolutionary ecology. In experiments, more heterozygous *V. americana* MLGs produced more turion biomass (Engelhardt et al. 2014) and larger turions produce larger plants with a greater number of associated ramets and turions. Such differential turion production could have yielded the dominant clones. At the same time, the ability of neutral processes to yield extensive clonal dominance after colonization has been demonstrated through modeling (Rafajlovic et al. 2017), and growth of heterozygous clones is not always favored after disturbance (Reusch 2006). Thus, it is possible that dominance by particular MLLs could result by chance if, over time, the MLLs that remained upstream happened to have higher levels of heterozygosity and served as the only source of propagules. Therefore, testing the acclimation potential for the widespread, dominant MLLs is of utmost importance for understanding whether active intervention is necessary for future resilience (Leimu et al. 2006). If the widespread and dominant MLLs outperform other MLLs in many different conditions across many sites, then increasing the genotypic diversity within the nontidal Potomac River may not be necessary and could even disrupt locally adapted gene complexes that are successful in this river. On the other hand, if MLLs became dominant through neutral processes, their potential for acclimation to novel conditions may be limited.

Potential for resilience also depends on whether the levels of diversity we observed result from long‐term processes or recent anthropogenic activities. If *V. americana* has survived for millennia with the observed levels of genotypic diversity and sexual reproduction, it is hard to argue that there have been dire consequences for resilience. By contrast, if the relatively few genotypes represent chance survivors of recent major anthropogenically driven declines that happen to persist in current conditions, the future risk of reduced resilience could be high.

### 4.4 Implications for Restoration

Given uncertainty in how the genetic structure in the Potomac River has come to be and about its impact on acclimation potential, we suggest that increasing genotypic diversity in the nontidal Potomac through genetic rescue would be a prudent action. Without intervention, increases in diversity are unlikely because large extents of single clones limit the proximity of different sexes and further favor clonal expansion due to the limited opportunities for sexual reproduction, and there is no nearby source of novel clonal diversity. Conservation geneticists increasingly suggest genetic rescue to be a priority management strategy in populations that are small, isolated, or drastically reduced (Broadhurst et al. 2008; Fenster and Dudash 1994; Ralls et al. 2018; Tallmon 2017; Weeks et al. 2011; Whiteley et al. 2015). However, it is usually suggested for overcoming risks from inbreeding (Broadhurst et al. 2008; Frankham 2015; Weeks et al. 2011), which is not an issue in the nontidal Potomac River. Rather, genetic diversity is limited by the small number of closely related MLLs that dominate across hundreds of kilometers, which imposes extremely small effective population sizes even with high stem counts. Low genotypic diversity also has implications for ecologically important factors such as the length of flowering, the presence of both sexes, and variation in vegetative reproduction versus flowering (Engelhardt et al. 2014). One expedient way to increase genotypic diversity would be to plant MLLs of the opposite sex into areas dominated by one MLL. This could be accomplished by propagating MLLs of known sex in isolation and transplanting ramets and turions into the field. An alternative approach that avoids the labor‐intensive propagation of MLLs would be to propagate locally collected male and female MLLs together and plant the resulting fruits and seeds directly in low diversity beds following proven methods (Moore and Jarvis 2007). Although most of the nontidal part of the river could benefit from increasing diversity, emphasizing upstream locations would provide a diversity of propagules to fuel downstream dispersal of seeds and vegetative propagules.

By contrast, in tidal sites there is no immediate imperative to increase genetic diversity. Still, managers seek to increase the acreage of submersed aquatic plants in this part of the Potomac River given their coverage remains below historic levels and targets set for the management of the Chesapeake Bay and its tributaries (Patrick et al. 2023; U.S. Environmental Protection Agency 2010). As submersed aquatic vegetation is one of the metrics for the health of the bay, ongoing low coverage contributes to poor grades in annual health report cards for the Bay (Vargas‐Nguyen et al. 2023). Managing nutrients and sediment and alleviating other contemporary anthropogenic pressures yield more growth and reproduction (Lefcheck et al. 2018; Zhang et al. 2018) than restoration plantings, which are often unsuccessful (Tanner et al. 2010). Further, annual monitoring data (Patrick et al. 2023) show that declines in water quality can rapidly reverse acreage gains achieved from active restoration. Still, active planting can provide founder colonies where no source populations remain.

Any active restoration effort requires choosing propagule source locations that reduce risks while fostering potential benefits. The temptation of moving propagules from one tributary to another within and across estuaries is great. Yet, it is important to reflect the natural biological processes that have shaped biodiversity patterns over millennia. Past efforts to restore *V. americana* have reflected levels of genotypic and allelic diversity and heterozygosity found in natural populations in the Chesapeake Bay, but not always the composition of genotypes or alleles (Lloyd et al. 2012). To better match genetic composition, Marsden et al. (2022) have suggested selecting *V. americana* propagules from sites in ecologically similar habitats (following suggestions of Giencke et al. 2018) that are within distances that interact through gene flow, or did so before fragmentation (Coart et al. 2005). Such sites are considered more likely to maintain local adaptation while allowing mixing of gene pools within a specified provenance (Breed et al. 2018). In *Zostera marina* , seeds harvested from nearby beds can preserve genetic diversity in restored sites with no signs of inbreeding depression in either donor or restored sites (Reynolds, Waycott, et al. 2012). We have evidence of local adaptation within different parts of the Chesapeake Bay (Engelhardt et al. 2014). However, the success of mixing MLGs varied by MLG and site and was not well predicted by levels of relatedness or differentiation (Marsden et al. 2013). Similarly, Reynolds et al. (2021) found variation in ecologically important traits across *V. americana* when samples from different Florida populations were exposed to stressful experimental conditions, but the responses were not predicted by the in situ conditions.

The dispersal distances and genetic breaks we identified using genetic markers provide guidance for these provenances. First, we have found different spatial structures of diversity in different rivers in eastern North America (Marsden et al. 2022), and between Potomac and Chesapeake Bay populations. At the same time, the risk of outbreeding depression was low for *V. americana* in the Bay (Marsden et al. 2013). Differences in connectivity observed in this study indicate that the spatial scale over which propagules should be moved differs dramatically between the nontidal and tidal reaches of the Potomac, and we therefore recommend different restoration strategies for the same species in the same river depending on the tidal regime:

1. Propagules from nontidal reaches should not be used in tidal reaches and vice versa given that the two tidal regimes have strikingly different genetic structures.
2. Because gene flow in the nontidal Potomac is high, we argue there is low risk in moving propagules among sites in that stretch of the river. If active restoration is desired, we recommend planting genotypes of the opposite sex that are currently most abundant in the area targeted for restoration, or by planting seeds as techniques are well established (Moore and Jarvis 2007). If harvesting in situ is not feasible, greenhouse propagation of different genotypes and crossing male and female genotypes is feasible.
3. We suggest using propagules from nearby locations in the tidal Potomac to maintain the genetic composition and structure of this tributary as a separate genetic population from the rest of the Bay (Lloyd et al. 2011). The harvest of ramets in the summer and seedpods in the fall is commonly used by managers but should only be attempted when the existing *V. americana* bed is healthy. A nursery of local genotypes could be useful to generate turions as well as seeds of known origin.

Restoration of submersed aquatic plant beds is of great interest worldwide given declining populations (Capistrant‐Fossa and Dunton 2024; Orth, Carruthers, et al. 2006; Waycott et al. 2009). Offsetting the multiple impacts of anthropogenic pressures in the Chesapeake Bay has been a key management goal for decades (Orth et al. 2017, 2010; Orth and Moore 1983, 1984) and climate change impacts only heighten ongoing concerns (Richardson et al. 2018). Active restoration is one of the practices often employed. Combined with past studies in which we found differences in genetic structure between the Potomac River and the Chesapeake Bay (Lloyd et al. 2011) and among North American rivers (Marsden et al. 2022), we show that there is no one‐size‐fits‐all strategy for *V. americana* . The magnitude of differences in different environments in the same river shows the challenges in extrapolating genetic data from one location to another. The data presented here contribute information that allows managers to incorporate eco‐evolutionary processes into restoration practices to yield a resilient future.

## Author Contributions

**Maile C. Neel:** conceptualization (equal), data curation (equal), formal analysis (equal), investigation (equal), methodology (equal), supervision (lead), visualization (equal), writing – original draft (supporting), writing – review and editing (lead). **Brittany W. Marsden:** conceptualization (equal), data curation (equal), formal analysis (equal), funding acquisition (lead), investigation (equal), methodology (equal), visualization (equal), writing – original draft (lead), writing – review and editing (supporting). **Katharina A. M. Engelhardt:** conceptualization (equal), writing – original draft (supporting), writing – review and editing (supporting).

## Conflicts of Interest

The authors declare no conflicts of interest.

## Data Availability

Raw genotype data and additional analysis files are available for reviewers on Dryad under the DOI: 10.5061/dryad.nvx0k6f17. All scripts are provided in an Rproject that is provided in a Zenodo archive under the DOI: 10.5281/zenodo.14787930. All data are provided within that RProject in addition to being given separately on Dryad.

## Appendix A

**TABLE A1:** *Vallisneria americana* collection details for 36 sites sampled from the Potomac River in Maryland, the District of Columbia, and Virginia, USA.

Reach	Site	Code		Old code^a^	Latitude	Longitude	River Km	Year sampled
Non tidal	Town Creek	1	TC	—	39.5227	−78.5399	462	2011
	Purslane Run	2	PR	—	39.5347	−78.4640	454	2011
	Fifteenmile Creek Upriver	3	FMC1	—	39.6241	−78.3848	417	2011
	Fifteenmile Creek Downriver	4	FMC2	TOUR1	39.6285	−78.3833	417	2007/08
	Sideling Hill Creek	5	SHC	TOUR2	39.6340	−78.3234	409	2007/08
	Sir John's Run	6	SJR	—	39.6516	−78.2399	399	2011
	Hancock Upriver	7	HCK1	—	39.6971	−78.1815	391	2011
	Hancock Downriver	8	HCK2	HCK	39.6974	−78.1767	391	2007/08
	Licking Creek	9	LC	—	39.6506	−78.0511	378	2011
	McCoy's Ferry	10	MF	—	39.6079	−77.9688	367	2011
	Williamsport Upriver	11	WSP1	WSP	39.6053	−77.8328	344	2007/08
	Williamsport Downriver	12	WSP2	—	39.6018	−77.8294	344	2011
	Opequon Creek	13	OJ	—	39.5155	−77.8606	330	2011
	Taylor's Landing	14	TL		39.4990	−77.7682	313	2011
	Snyder's Landing	15	SL	—	39.4991	−77.7682	307	2011
	Antietam Creek	16	AC	—	39.4200	−77.7481	295	2011
	Brunswick Upriver	17	BWK1	BWK	39.3062	−77.6164	270	2007/08
	Brunswick Downriver	18	BWK2	—	39.3063	−77.6151	270	2011
	Point of Rocks Upriver	19	POR1	POR	39.2727	−77.5416	262	2007/08
	Point of Rocks Downriver	20	POR2	—	39.2701	−77.5307	262	2011
	White's Ferry Upriver	21	WF1	WF	39.1556	−77.5195	241	2007/08
	White's Ferry Downriver	22	WF2	—	39.1550	−77.5196	241	2011
	Edward's Ferry	23	EF	—	39.1030	−77.4738	233	2011
	Pennyfield Lock	24	PL	PL	39.0533	−77.2911	216	2007/08
Tidal	George Washington Parkway	25	GWP	GWP	38.7303	−77.0416	163	2007/08
	Piscataway Park	26	SWP	SWP	38.6849	−77.1019	156	2007/08
	Pohick Bay	27	PB	—	38.6770	−77.1687	150	2013
	Gunston Manor	28	GM	GM	38.6353	−77.1441	146	2007/08
	Belmont Bay A	29	BB_A	—	38.65627	−77.2233	142	2013
	Belmont Bay B	30	BB_B		38.64699	−77.2225	142	2013
	Leesylvania State Park	31	LSP	LSP	38.5835	−77.2583	138	2007/08
	Mattawoman Creek	32	MWC	—	38.57137	−77.1807	135	2013
	Aquia Landing	33	AL	AL	38.3884	−77.3213	114	2007/08
	Nanjemoy Creek	34	NC	—	38.46403	−77.141	95	2013
	Port Tobacco River A	35	PTR_A		−77.0239	38.49631	89	2013
	Port Tobacco River B	36	PTR_B	—	−77.0265	38.48449	89	2013

^a^ Site names of samples collected in 2007–2008 and described in Lloyd et al. (2011).

## Appendix B

**TABLE A2:** Nearest neighbor and maximum distances (m) among samples within sites.

Site		Nearest neighbor distance (m)			Maximum distance (m)		
		Minimum	Median	Maximum	Minimum	Median	Maximum
1	TC	1.9	9.4	22.2	107.4	144.3	192.8
2	PR	4.3	10.2	14.7	102.7	151.9	194.7
3	FMC1	1.9	10.2	91.1	226.2	353.2	438.9
4	FMC2	6	8.2	12.5	66.6	98	129.2
5	SHC	0	13.7	19.8	108.4	154.6	203
6	SJR	1.9	14.2	38.6	317.1	435.6	594.3
7	HCK1	2.3	7.8	25.8	111.5	153.6	192.7
8	HCK2	0	12	56.1	237.6	314.8	444.6
9	LC	4.7	13	57.7	295.4	486.2	555.4
10	MF	3.4	7.9	18.7	167.6	261.4	329.3
11	WSP1	0	12	25.5	297.6	447.3	522.6
12	WSP2	0	6.2	25	170.5	246.9	327.9
13	OJ	1.8	7.2	90.2	255.9	357	472.7
14	TL	12	16.6	77.3	77.3	104.7	153.6
15	SL	4.7	10.8	24.1	109.2	156	216.1
16	AC	4	12.2	67.6	316.2	517.5	628.6
17	BWK1	12.4	21.9	32.7	231.3	342.4	452.2
18	BWK2	2.3	7.8	28.6	177.9	255.7	355
19	POR1	0	11.7	39.8	330.7	528.7	645.3
20	POR2	3.4	12.5	41.7	192.8	264.2	381.1
21	WF2	1.9	13.2	73.1	372.4	582.6	717
22	WF1	6.5	13.7	52.3	422.1	534.6	717.8
23	EF	2.3	11.8	35.2	238.1	328.8	450.5
24	PL	10.4	15	26.3	275.7	415.2	538.7
25	GWP	3.4	6.1	16.7	107.7	160.4	213.1
26	SWP	4.8	5.6	21.2	121.3	175.5	240.3
27	PB	4.8	13.8	76.2	292.0	383.3	403.7
28	GM	3.7	4.7	12.4	111.8	174	215
29	BB_A	1.1	4.2	137.1	591.6	839.5	1027.1
30	BB_B	1.1	6.2	31	342.6	537.5	660.6
31	LSP	0	8.2	11.1	121.1	187	235.5
32	MWC	6.5	27.9	59	2994.5	4410.7	4651.8
33	AL	6.5	8.5	10.2	139.8	206.9	268.9
34	NC	0.9	12.7	67.7	1990.2	2903	3105.5
35	PTR_A	7.1	15.8	92.8	525.8	630.7	826.9
36	PTR_B	8.7	19.2	70.9	575.4	619.1	706.2
	Median	3.4	11.7	32.7	231.3	328.8	444.6
	Minimum	0	4.2	10.2	66.6	98	129.2
	Maximum	12.4	27.9	137.1	2994.5	4410.7	4651.8
